# Genome-wide association study of yield and related traits in common wheat under salt-stress conditions

**DOI:** 10.1186/s12870-020-02799-1

**Published:** 2021-01-07

**Authors:** Pan Hu, Qi Zheng, Qiaoling Luo, Wan Teng, Hongwei Li, Bin Li, Zhensheng Li

**Affiliations:** State Key Laboratory of Plant Cell and Chromosome Engineering, Institute of Genetics and Developmental Biology, The Innovative Academy of Seed Design, Chinese Academy of Sciences, Beijing, 100101 China

**Keywords:** Common wheat, Favorable allele, GWAS, Salt stress, Wheat660K SNP chip, Yield-related traits

## Abstract

**Background:**

Soil salinization is a major threat to wheat production. It is essential to understand the genetic basis of salt tolerance for breeding and selecting new salt-tolerant cultivars that have the potential to increase wheat yield.

**Result:**

In this study, a panel of 191 wheat accessions was subjected to genome wide association study (GWAS) to identify SNP markers linked with adult-stage characters. The population was genotyped by Wheat660K SNP array and eight phenotype traits were investigated under low and high salinity environments for three consecutive years. A total of 389 SNPs representing 11 QTLs were significantly associated with plant height, spike number, spike length, grain number, thousand kernels weight, yield and biological mass under different salt treatments, with the phenotypic explanation rate (*R*^2^) ranging from 9.14 to 50.45%. Of these, repetitive and pleiotropic loci on chromosomes 4A, 5A, 5B and 7A were significantly linked to yield and yield related traits such as thousand kernels weight, spike number, spike length, grain number and so on under low salinity conditions. Spike length-related loci were mainly located on chromosomes 1B, 3B, 5B and 7A under different salt treatments. Two loci on chromosome 4D and 5A were related with plant height in low and high salinity environment, respectively. Three salt-tolerant related loci were confirmed to be important in two bi-parental populations. Distribution of favorable haplotypes indicated that superior haplotypes of pleiotropic loci on group-5 chromosomes were strongly selected and had potential for increasing wheat salt tolerance. A total of 14 KASP markers were developed for nine loci associating with yield and related traits to improve the selection efficiency of wheat salt-tolerance breeding.

**Conclusion:**

Utilizing a Wheat660K SNPs chip, QTLs for yield and its related traits were detected under salt treatment in a natural wheat population. Important salt-tolerant related loci were validated in RIL and DH populations. This study provided reliable molecular markers that could be crucial for marker-assisted selection in wheat salt tolerance breeding programs.

**Supplementary Information:**

The online version contains supplementary material available at 10.1186/s12870-020-02799-1.

## Background

The arable land affected by salinity worldwide is greater than 800 million ha, which is analogous to ~ 6% of the global land area [1–3]. Moreover, the proportion of salt-affected soil is increasing annually owing to irrigation, which could lead to salt accumulation in agricultural soils [1, 4]. Therefore, salinization has become a major limitation of grain yield [2]. Common wheat (*Triticum aestivum* L.), an important cereal crop, provides calorific energy for ~ 20% of the world population (http://www.fao.org/faostat). A significant increase in wheat yield is required to meet the food needs of the growing world population [5, 6]. Common wheat is a moderately salt-tolerant crop and can produce a reduced yield when grown in saline soil [1, 6, 7]. Therefore, it is critical to understand the salt-stress response- and adaptation-related mechanisms in common wheat and to utilize the salt tolerant varieties in wheat breeding and application of genetic resource.

Some salinity adaptation-related mechanisms, such as osmotic adjustment, tissue-tolerance processes and Na^+^ exclusion, have been elaborated in rice, maize and barely [1, 2, 8, 9]. However, studies of salinity tolerance in wheat are limited owing to its large (~ 17 Gb), complicated and hexaploid (AABBDD) genome [10, 11]. Notably, with the development of high-throughput sequencing technology, increasing whole genome-based information has been released for wheat [12]. Simultaneously, many single nucleotide polymorphisms (SNPs) were identified and placed on SNP chips, which have become powerful genotyping platforms in genome-wide association studies (GWASs) and quantitative trait loci (QTL) mapping [5]. For instance, QTLs that control grain shape and size were revealed by Wu et al. [13] in wheat Yanda1817/Beinong6 recombinant inbred lines (RILs) using the wheat Infinium iSelect 9 K SNP genotyping assay. Ma et al. [14] performed a GWAS for six yield-related traits in a breeding population derived from Xiaoyan 6 using the wheat 90 K array. A range of diploid, tetraploid and hexaploid wheat accessions were characterized by a high density wheat 820 K array, and a wheat consensus map was constructed using 56,505 SNP markers [15]. Recently, a new wheat SNP array, Wheat660K, was designed by the Chinese Academy of Agricultural Sciences. Wheat660K array contains ~ 630,517 SNPs generated from the low coverage sequencing of 192 common wheat and related species accessions (https://wheat.pw.usda.gov/ggpages/topics/Wheat660_SNP_array_developed_by_CAAS.pdf). With the advantages of genome specificity and high density, the Wheat660K array has a wide range of possible applications in gene discovery, haplotype mapping, genomic selection and evolutionary studies. For example, a major effect QTL that might be a new adult plant-resistance gene to stripe rust was detected in wheat cultivar Yaco “S” using the Wheat660K SNP chip [16]. Cui et al. [11] characterized a major stable QTL for kernel number per spike on a high-density genetic map of hexaploid wheat using the Wheat660K array. Markers that are closely linked to the dwarfing gene *Rht24* were identified by Tian et al. [17] utilizing the Wheat660K array.

GWASs are based on linkage disequilibrium, and the method has become a popular and efficient tool for gene discovery and marker development for complex traits [18]. With several advantages, such as the use of diverse germplasms as materials and the capability to capture superior alleles that have been missed by breeding practices, GWASs have been widely used to dissect complex genetic mechanisms regulating biotic and abiotic stress tolerance in wheat [16, 19, 20]. For instance, Riaz et al. [21] performed a GWAS using 10,748 DArT-seq markers in a diverse panel of 295 bread wheat accessions for leaf rust resistance. Mwadzingei et al. [22] assessed the various agronomic traits of 93 diverse bread wheat genotypes under drought-stress conditions. However, limited research to identify major stable loci of wheat under salt-stress conditions has been conducted using GWASs. Hussain et al. [23] identified QTLs linked with micronutrient concentrations in wheat F_2_ lines under salt-stress conditions at the seedling stage. Salt tolerance QTLs have been identified in winter wheat cultivars at germination and seedling stages using GWASs [20]. Furthermore, most previous studies were confined to the wheat seedling stage. Limited studies focused on the wheat adult stage.

Here, the performances of 191 wheat accessions were evaluated during three consecutives growing seasons at Nanpi eco-agricultural experimental station, which possesses the typical salinization soil in the low plain around Bohai Sea. And Wheat660K array was used to identify SNP markers associated with eight phenotypic traits of the wheat accessions under different salinity conditions, with the aims of providing valuable information on important allelic variations affecting wheat yield and related traits under practical salt-stress conditions and finding usable SNP markers and varieties for wheat salt-tolerance breeding programs.

## Results

### Responses to salinity stress, variations and correlations between phenotypic traits

The phenotypes of 191 wheat accessions were characterized during three crop seasons (2014–2017) in low salinity (LS) and high salinity (HS) environments. Significant differences existed among accessions for all the traits in the two different salinity treatments. Compared with LS conditions, the mean values for the eight phenotypic traits decreased to different degrees in HS treatment, indicating more significant inhibitory effects on the growth of wheat accessions under HS conditions (Table 1). Additionally, correlation co-efficient analyses were conducted among the three field experiments for the same trait under the same treatment. All the adult-stage traits had highly significant positive correlations, implying the moderate repeatability for these traits in field experiments (Table S2). Estimations of heritability for all the phenotypic traits showed moderate to high heritability levels. All the phenotypic traits showed higher heritability levels under LS conditions than under HS conditions (Table 1). PH showed the highest heritability levels under LS (*H*^*2*^ = 88.58%) and HS (*H*^*2*^ = 56.30%) conditions. SL and KPS have moderate and relatively stable heritability levels, with *H*^*2*^ values of 56.83 and 39.5% under LS conditions and 46.52 and 31.59% under HS conditions, respectively. Compared with the other traits, YPP and GN showed similar but lower heritability level under LS and HS conditions (Table 1). Pearson’s co-efficient of correlation between traits was calculated based on data averaged across 3 years under the two salinity conditions (Table 2). PH showed significantly positive correlations with SN, SL, KPS, BM, YPP and GN under both salt-stress conditions, and the correlation coefficients between PH and both BM and GN were comparatively higher than those between PH and other traits in the two salinity environments. TKW showed significantly negative correlations with PH, SN, KPS and GN, but was positively correlated with SL and YPP under both salinity treatments. In addition, BM and GN showed extremely high and positive correlations with YPP under LS and HS conditions. The correlations between yield and its components (SN, KPS and TKW) were significantly positive under both salinity conditions. Importantly, the correlation coefficients between YPP and SN were highest under both conditions, while the correlation coefficients between YPP and TKW were lower, compared with those between YPP and KPS. Interestingly, compared with LS treatment, the correlation coefficients between YPP and both SN and KPS slight increased under HS treatment.

**Table 1:** Descriptive statistics and variance parameters estimated for eight traits studied on 191 wheat accessions under low and high salinity conditions from 2015 to 2017

		Descriptive		Variance parameters					
		Mean	Range	G	G × E	E	*H*^*2*^ (%)		
PH (cm)	LS	76.1	49.80–149.00	85.3	2.6	2.52	88.58		
	HS	63.89	21.00–118.00	48	12.2	22.1	56.3		
SN	LS	12.63	2.00–33.00	24.1	16.9	7.98	29.62		
	HS	8.8	1.00–30.00	15.5	38.1	9.55	25.65		
SL (cm)	LS	8.25	0.50–14.00	34.9	38.4	4.38	56.83		
	HS	7.87	3.50–13.20	35.2	24.1	17.5	46.52		
KPS	LS	49.77	18.00–108.00	33.8	13.3	7.15	39.5		
	HS	49.03	10.00–109.00	30.5	2.65	21.2	31.59		
BM (g)	LS	45.17	4.00–109.48	26.9	6.35	10.2	29.48		
	HS	30.01	0.72–91.72	14.3	33.1	12.5	21.95		
YPP (g)	LS	19.87	2.30–52.07	25.3	0.37	11.6	26.02		
	HS	14.03	0.31–41.97	17.2	17.6	14.3	21.18		
GN	LS	486.94	54.00–1342.00	21.6	18.8	9.7	27.56		
	HS	344.23	31.00–1360.00	18.2	24.6	14.3	24.4		
TKW (g)	LS	41.77	15.62–71.96	25.1	57.6	8.09	60.03		
	HS	41.39	7.80–63.65	34.9	26.1	13.9	47.86		

*PH* plant height, *SN* spike number, *SL* spike length, *KPS* kernels per spike, *GN* grain number, *TKW* thousand kernels weight, *YPP* yield per plot, *BM* biological mass, *G* genotype, *E* environment, *G × E* genotype×environment, *H*^*2*^ broad-sense heritability

**Table 2:** Correlation analysis of different traits for 191 common wheat accessions under low and high salinity treatments

	PH	SN	SL	KPS	BM	YPP	GN	TKW	
PH		0.494^**^	0.405^**^	0.362^**^	0.510^**^	0.415^**^	0.553^**^	−0.358^**^	PH
SN	0.335^**^		−0.08	0.028	0.811^**^	0.785^**^	0.891^**^	−0.162^*^	SN
SL	0.284^**^	−0.079		0.331^**^	0.282^**^	0.260^**^	0.164^*^	0.151^*^	SL
KPS	0.314^**^	−0.093	0.262^**^		0.327^**^	0.317^**^	0.398^**^	−0.303^**^	KPS
BM	0.496^**^	0.764^**^	0.164^*^	0.285^**^		0.964^**^	0.897^**^	0.12	BM
YPP	0.303^**^	0.740^**^	0.156^*^	0.238^**^	0.941^**^		0.889^**^	0.205^**^	YPP
GN	0.447^**^	0.859^**^	0.032	0.356^**^	0.870^**^	0.855^**^		−0.218^**^	GN
TKW	−0.308^**^	−0.227^**^	0.223^**^	−0.271^**^	0.079	0.215^**^	−0.293^**^		TKW

The upper triangular matrix represents high salinity; the lower triangular matrix represents low salinity

*PH* plant height, *SN* spike number, *SL* spike length, *KPS* kernels per spike, *GN* grain number, *TKW* thousand kernels weight, *YPP* yield per plot, *BM* biological mass

*represents significant difference determined at *p*<0.05, ** represents significant difference determined at *p*<0.01

### SNP genotyping

Based on the quality preprocessing results provided by PLINK software (http://zzz.bwh.harvard.edu/plink/data.shtml), 8451 and 221,878 SNPs were removed for having call rates less than 80% and minor allele frequencies less than 0.05, respectively. No accession was removed for low genotyping (MIND > 0.1). Finally, 191 accessions containing 387,657 SNPs were retained for further analyses. And among these, 322,590 SNP markers were mapped on the Wheat660K consensus maps (Prof.Jizeng Jia, pers.comm.). The numbers of SNPs presented on each wheat chromosome were provided in Table S3. In total, 740,124 variation-containing alleles were detected by 387,657 SNPs among the 191 cultivars (Table S3). The range of the mean allele numbers on the 21 wheat chromosomes was from 1.38 to 3.25. The greatest mean allele numbers were on chromosomes 6D, 2D and 4A, successively. Chromosome 3B had the lowest mean allele number (Table S3).

### Genetic structure and linkage disequilibrium

**Fig. 1:**
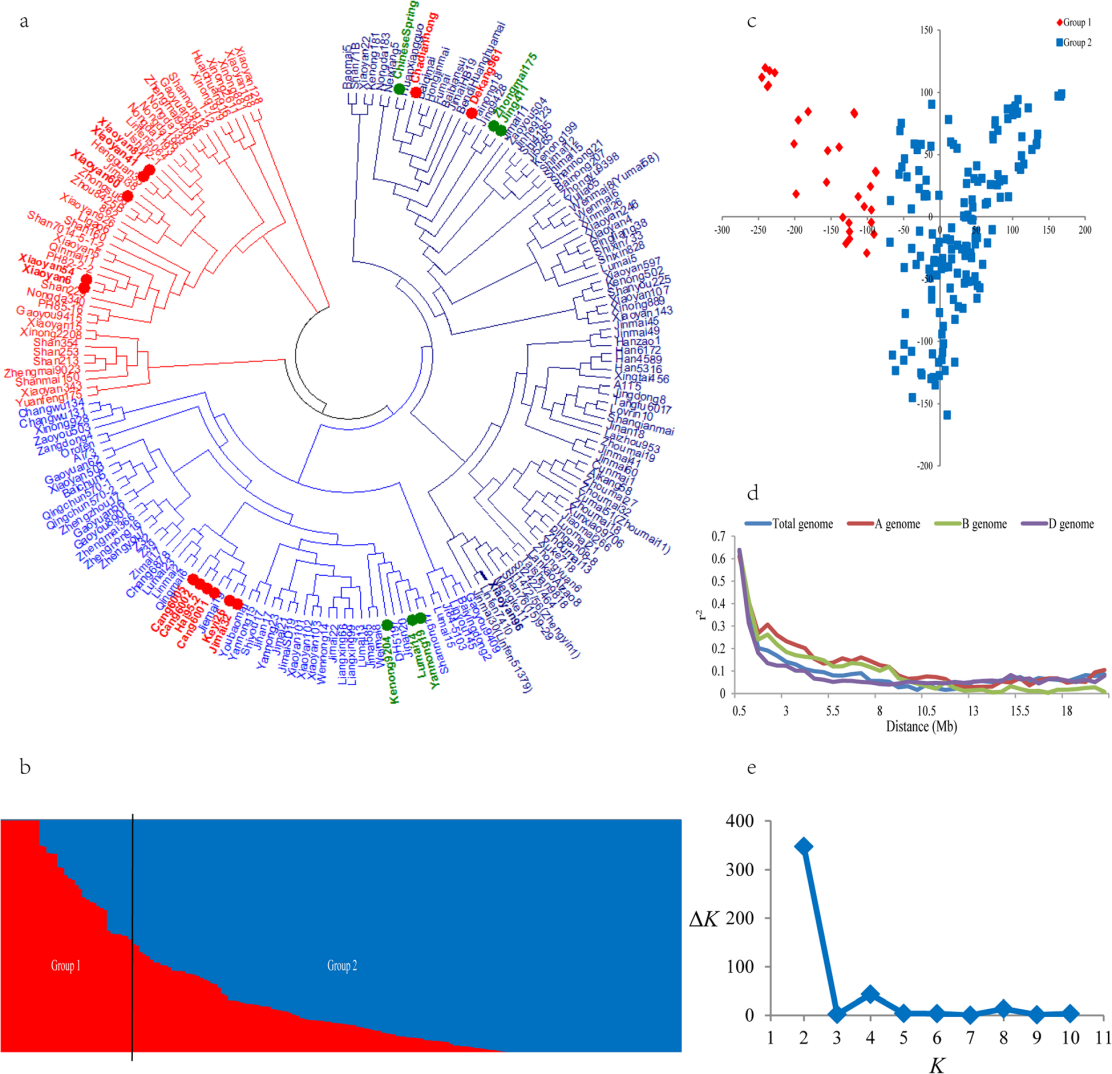
Population structure of the common wheat population. **a** Neighbor-joining tree analysis of 191 wheat accessions. **b** Stacked bar plot of ancestry relationship of 191 wheat accessions when *K* = 2. **c** Plot of the first principle component against second principle component. **d** Decay of linkage disequilibrium (LD) in the A, B, D sub-genomes and total genome of the common wheat population. **e** Plot of delta *K* against putative *K* ranging from 1 to 10

Different methods were used to analyze the population structure. The neighbor-joining method based on Nei’s standard genetic distance [24] was used to classify the 191 accessions, and it indicated that they were divided into two groups (Fig. 1a). The first group (45) mainly consisted of improved varieties from Shaanxi. The second group (146) was mainly comprised of improved varieties originating from Beijing, Hebei, Henan and Shandong. These two groups were further confirmed by PCA and population structure analyses. When the number of subpopulations (*K*s) was plotted against the Δ*K* calculated using software Structure, the highest Δ*K* was observed at *K* = 2 (Fig. 1b and e), indicating that the 191 accessions could be divided into two subgroups. The PCA also showed two distinct clusters (Fig. 1c). According to the phylogenetic tree constructed using the neighbor-joining method, the well-known salt-tolerant wheat accessions, such as Cang 6001, Cang 6002, Cang 6005, Dekang 961, Jimai 32 and Keyi 26, clustered into group 2.

Linkage disequilibrium was calculated using 153,725, 184,755, and 49,177 SNP markers for the A, B, and D sub-genomes, respectively. Based on the critical *r*^*2*^ threshold value at 0.1, the LD decay distance of the whole genome was approximately 5.0 Mb. The highest LD decay distance of 8.5 Mb was found in the A sub-genome, followed by 8.0 Mb in the B sub-genome. The D sub-genome has the lowest LD decay distance of 3.5 Mb (Fig. 1d).

### Associations between traits and SNPs

**Fig. 2:**
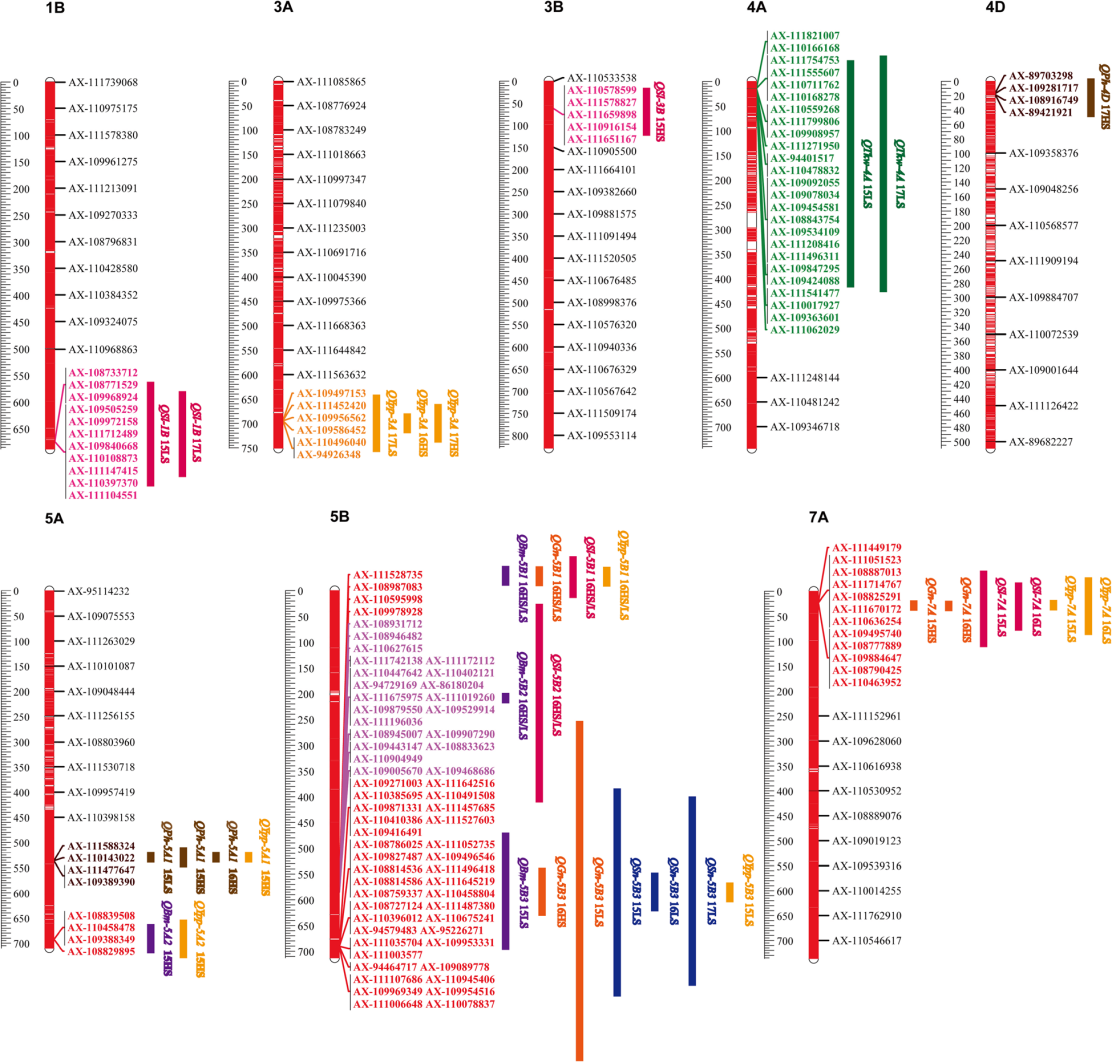
Distributions of major QTLs for phenotype traits on chromosomes in 191 common wheat accessions. Bars with brown, blue, rose red, violet, yellow, orange and green colors represents plant height (PH), spike number (SN), spike length (SL), biological mass (BM), yield per plot (YPP), grain number (GN), and thousand kernels weight (TKW), respectively. The length of bars indicates the number of SNPs for detected QTL. *QPh* represents QTL for PH, *QSn* represents QTL for SN, *QSl* represents QTL for SL, *QBm* represents QTL for BM, *QYpp* represents QTL for YPP, *QGn* represents QTL for GN, *QTkw* represents QTL for TKW. LS indicates low salinity treatment; HS indicates high salinity treatment

Association analyses were performed between the phenotypic traits at the adult stage and SNP markers. In total, 611 significantly associated signals among 389 SNPs were identified for eight phenotypic traits in the six environments at *P <* 1.25e-6 (Table S4). The phenotypic variation explanation rate (*R*^2^) ranged from 9.14 to 68.81% (Table S4). A total of 152 SNPs showed significant associations with the same trait in two or more environments, and 44 SNPs showed significant associations with two or more traits (Table S5). In general, 11 significantly associated loci (a 5 Mb interval containing three or more significant associated SNPs was regarded as a locus) that might affect the growth of wheat under salt-stress conditions were among the 389 SNPs associated with PH, SL, SN, YPP, GN, BM and TKW (Fig. 2).

### Plant height

Up to 141 significant SNPs associated with PH were detected and mainly distributed on chromosomes 2B, 2D, 3D, 4A, 4D, 5A, 6B, 7B and 7D (Table S4). Of these, 79 SNPs were detected in at least two environments under LS conditions and were mainly located on chromosome 5A (Table S5). Significant associations were detected in one environment under HS conditions for eight SNPs related to PH, which were located on chromosome 4A (2), 4D (4) and 5A (2) (Table S5). One SNP locus named *QPh-5A1*, which contained four SNPs and was located on chromosome 5A at 535.91–536.80 Mb, was significantly associated with PH in 15LS and 15HS, explaining 44.46–67.67% of the phenotypic variation. More importantly, one SNP of *QPh-5A1* was significantly associated with YPP in 15HS. On chromosome 4D, four SNPs located in the interval 16.93–19.29 Mb (Fig. 2), defined as *QPh-4D*, were associated with PH in 17HS and explained 52.81–53.62% of the phenotypic variation.

### Spike length

A total of 80 significant SNPs were associated with SL and distributed on chromosomes 1A, 1B, 2A, 2D, 3B, 3D, 5B, 5D, 6D and 7A (Table S4). Among them, nine SNPs on chromosome 1B and three SNPs on 7A were detected in two environments under LS conditions (Table S5). Meanwhile, four SNPs on chromosomes 1A (1), 1B (1), 2A (1) and 2D (1) were detected in two environments under HS conditions (Table S5). The important loci associated with SL were mainly located on chromosomes 1B, 3B, 5B and 7A based on the repeatability scores. Locus *QSl-1B*, including 11 significant SNPs in 15LS and 17LS, was located at 674.96–675.13 Mb on chromosome 1B (Fig. 2) and explained 16.38–23.12% of the phenotypic variation. Locus *QSl-3B*, consisting of five SNPs, was associated with SL in 15HS and located at 62.13–62.14 Mb on chromosome 3B (Fig. 2) and explained 12.18–12.82% of the phenotypic variation. On chromosome 5B, four SNPs located at 574.06–578.35 Mb (Fig. 2), named as *Q-5B1*, were associated with the relative value of SL (16HS/16LS) and explained 12.94–14.29% of the phenotypic variation. Importantly, two SNPs of *Q-5B1* were significantly associated with the relative values of BM, GN and YPP. Locus *QSl-5B2*, containing 21 SNPs, was located in the interval 583.48–584.89 Mb on chromosome 5B (Fig. 2). It was significantly associated with the relative value (16HS/16LS) of SL and could explain 13.03–15.28% of the phenotypic variation (Table S5). Furthermore, one SNP of *QSl-5B2* was significantly associated with the relative values (16HS/16LS) of BM (Table S5). Locus *Q-7A*, involving 12 SNPs, was mapped at 22.75–22.88 Mb on chromosome 7A (Fig. 2), among which ten SNPs were significantly associated with SL in 15LS and 16LS, explaining 20.20–28.47% of the spike length variation. In total, seven SNPs of *Q-7A* were significantly associated with YPP in 15LS and 16LS and two SNPs of *Q-7A* were significantly associated with GN in 15HS and 16HS (Table S5).

### Grain number

In total, 60 SNPs were significantly associated with GN and were mainly located on chromosomes 2A, 3A, 5B and 7A (Table S4). A comparison of the associated SNPs for SN and GN showed abundantly common SNPs from chromosome 5B that were accumulated at the common locus *Q-5B3* (690.08–690.70 Mb) (Fig. 2). At this locus, 34 SNPs were detected in LS environments for GN, and five SNPs were detected under both LS and HS conditions. Notably, the correlation analysis of pairwise traits showed extremely high co-efficient of correlation between GN and SN, with values were 0.859 and 0.891 under LS and HS treatments, respectively.

### Biological mass

Up to 18 significant SNPs were associated with BM, and they were mainly distributed on chromosomes 4A, 4B, 5A and 5B (Table S4). Of these, nine and three SNPs on chromosome 5B were detected in 15LS and 16HS/16LS and could explain 10.04–13.90% and 12.61–12.80% of the phenotypic variation, respectively.

### Yield per plant

A total of 48 significant SNPs were associated with YPP and were mainly distributed on chromosomes 2D, 3A, 5A, 5B and 7A (Table S4). Of them, six SNPs, forming locus *QYpp-3A*, were located on chromosome 3A at 694.01–694.96 Mb. The locus was associated with YPP in 16HS, 17LS and 17HS with a phenotypic variation of 11.36–16.32% (Fig. 2). In addition, four SNPs, composing locus *Q-5A2*, were located on chromosome 5A at 692.16–692.39 Mb and detected in 15HS, with a phenotypic variation of 10.69–12.70% (Fig. 2). The SNPs of *Q-5A2* were significantly associated with BM in 15HS. The common locus associated with YPP and SL was *Q-7A* (22.75–22.88 Mb), which was observed on chromosome 7A (Fig. 2).

### Spike number

As many as 68 significant SNPs were associated with SN and were mostly located on chromosomes 1A, 1B, 2A, 2B, 5A, 5B, 5D, 6D and 7B (Table S4). Of these, 19 SNPs on chromosome 5B were detected in at least two environments under LS conditions, while two SNPs on chromosome 5A were detected in at least two environments under HS conditions. Locus *Q-5B3* consisting of 36 SNPs was found on chromosome 5B at 690.08–690.70 Mb, and 24 SNPs in this locus were associated with SN in 15LS, 16LS and 17LS (Fig. 2) and could explain 17.29–32.82% of the phenotypic variation. Moreover, the SNPs of *Q-5B3* were almost significantly associated with not only GN in 15LS (34) and 16HS (5), but BM in 15LS (9) (Table S5).

### Thousand-kernel weight

Totally, 31 significant SNPs associated with TKW were mainly distributed on chromosomes 3B, 3D, 4A, 4B and 7B (Table S4). Among these, the largest number of SNPs (up to 25) was from chromosome 4A. These SNPs, forming locus *QTkw-4A* at 14.68–16.28 Mb, were detected in 15LS and 17LS and could explain 18.60–35.57% of the phenotypic variation.

### Phenotypic effects of alleles in the natural population

A *t* test was performed to verify the favorable alleles in the natural population based on the significantly associated SNPs, and some loci in this study were identified as possibly affecting the agronomic traits of wheat under salt-stress conditions. For instance, accessions with favorable alleles at loci *QSl-1B* (674.96–675.13 Mb), *QSl-3B* (62.13–62.14 Mb) and *QSl-5B2* (583.48–584.89 Mb) showed longer SLs (increased by 0.54–0.97 cm with LS and by 0.57–2.44 cm with HS). Favorable alleles of locus *QTkw-4A* (14.68–16.28 Mb) increased the TKW by 4.06–5.32 g under LS and by 2.49–5.95 g under HS. Favorable alleles of locus *QPh-4D* (16.93–19.29 Mb) increased PH by 3.42–4.57 cm under LS and by 4.21–4.31 cm under HS. Accessions with favorable alleles at locus *Q-5B1* (574.06–578.35 Mb) have longer SLs (by 1.06–2.46 cm) and greater BMs (by 4.72–5.45 g), GNs (by 23.80–65.29) and YPPs (by 2.58–8.69 g) under HS. Accessions with favorable alleles at locus *Q-5B3* (690.08–690.70 Mb) have greater BMs (by 1.49–4.54 g under LS and by 1.44–1.89 g under HS), SNs (by 0.46–1.50 under LS and by 0.35–0.64 under HS), GNs (by 14.49–60.75 under LS and by 16.16–25.68 under HS) and YPPs (by 2.71–5.73 g under LS and by 2.43–2.88 g under HS). Favorable alleles of locus *Q-7A* (22.75–22.88 Mb) increased SL and YPP by 0.71–1.28 cm and 2.16–3.39 g under LS, respectively, and increased SL by 1.07–1.11 cm under HS (Table S6).

The frequencies of the alleles of the above key loci ranged from 5.76 to 93.72% (Table S6). An analysis of the distributions of favorable alleles indicated that introduced varieties possessed the highest average number of favorable alleles, followed by improved cultivars. The average number of favorable alleles in landrace was the lowest (Fig. S1a). Except accessions from Gansu and Sichuan, which were mostly landraces, accessions from Hebei, Henan and Shaanxi had comparatively higher average numbers of favorable alleles, followed by the cultivars from Shandong, Beijing and Shanxi. However, cultivars from Qinghai had the lowest average number of favorable alleles (Fig. S1b). Thus, it was believed that the introduced varieties and the improved cultivars should be utilized to improve these yield-related traits in salinity tolerant wheat cultivars. In addition, the favorable alleles of the same associated trait varied for some well-known cultivars. For example, although accessions Kenong 181, Liangxing 99, Zaoyou 504 and Jinhe 9123 had similar SL, Kenong 181 and Liangxing 99 contained the favorable alleles from loci *QSl-1B*, *QSl-5B2* and *Q-7A*, whereas Zaoyou 504 and Jinhe 9123 had the favorable alleles from loci *QSl-1B*, *QSl-3B1*, and *Q-7A*. Besides, the YPP of landrace Chadianhong and Baiqimai was similar. Chadianhong contained the favorable alleles from loci *Q-5A2* and *Q-7A*, while Baiqimai had the favorable alleles from loci *QYpp-3A* and *Q-5A2*.

### Development of Kompetitive Allele Specific PCR (KASP) markers for the important loci

In total, 14 KASP markers were designed and used to detect polymorphisms in ZX-RIL and HL-DH populations (Table S8). Eight KASP markers were polymorphic. Of these, four (*kasp-5695*, *kasp-9337*, *kasp-6491* and *kasp-4516*) showed polymorphisms in ZX-RIL population, two (*kasp-9179* and *kasp-4767*) were polymorphic in HL-DH population, and two (*kasp-8827* and *kasp-9508*) were polymorphic in both ZX-RIL and HL-DH populations. Based on the genotyping results of each KASP marker, a *t* test was conducted for corresponding traits in the related DH and RIL populations.

KASP marker *kasp-9508*, which came from locus *Q-5A2*, was polymorphic in ZX-RIL as well as HL-DH populations. In HL-DH population, *kasp-9508* was significantly correlated with PH, BM and TKW in two environments (LS and HS) and was correlated with YPP under HS conditions (Fig. S2a–d). For the locus *Q-7A*, markers *kasp-9179* and *kasp-4767* were polymorphic in HL-DH population. *Kasp-9179* was significantly correlated with PH in two environments (LS and HS), with SL under LS conditions, and with BM and YPP under HS conditions (Fig. S2e–h). *Kasp-4767* was significantly correlated with PH, BM and YPP under HS conditions and with SL under LS conditions (Fig. S2i–l). Thus, loci *Q-5A2* and *Q-7A* should be stable and pleiotropic loci affecting BM, PH, SL, TKW and YPP under salt-stress conditions.

## Discussion

### Comparison with previous studies and gene prediction of key loci

In the present study, 191 wheat accessions were genotyped using the Axiom Wheat660K array, which were used to identify genomic regions and SNPs associated with agronomic traits under saline conditions using a GWAS. SNPs significantly associated with plant type, spike characteristics, grain yield and its component traits were mainly observed on chromosomes 1B, 3A, 3B, 4A, 4D, 5A, 5B, and 7A. Moreover, the number of significantly associated SNPs identified under LS conditions was much more than those under HS conditions, which was similar to the results obtained under drought-stress conditions [22, 25], indicating that genotype by environment interactions could highly influence the salinity tolerance capability of wheat cultivars. In total, 11 loci located on chromosomes 1B (1), 3A (1), 3B (1), 4A (1), 4D (1), 5A (2), 5B (3) and 7A (1) were identified to play important roles in modulating the agronomic traits of wheat cultivars under salt-stress (Table S6).

**Fig. 3:**
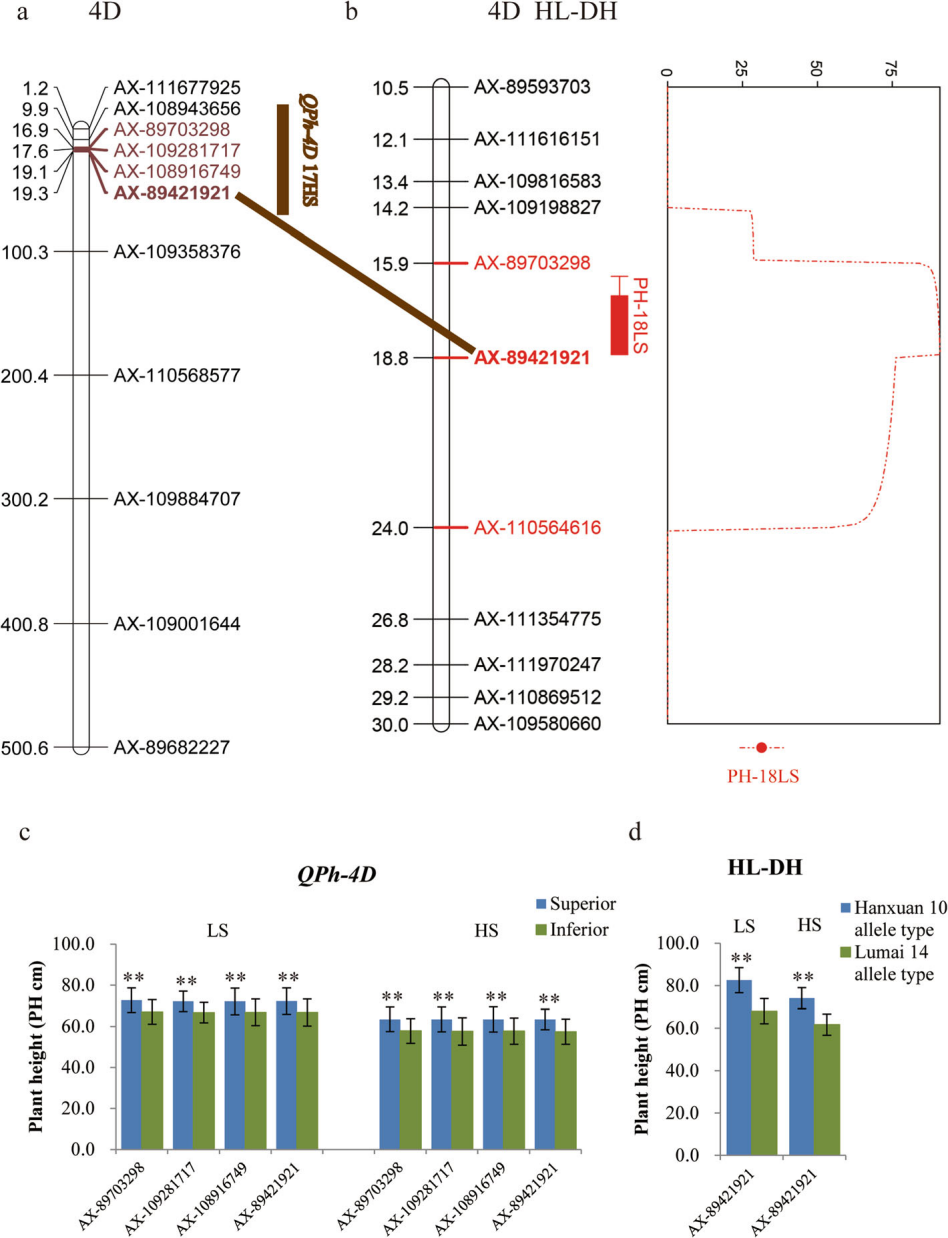
QTL position on chromosome 4D for plant height (PH) in the common wheat population and HL-DH population. **a** Chromosome location of *QPh-4D* in the 191 common wheat accessions. **b** QTL position for PH in DH population of “Hanxuan 10×Lumai 14”. **c** The phenotype values of accessions with superior allele of *QPh-4D* in the common wheat population. **d** The phenotype values of accessions with superior allele of *QPh-4D* in DH population of “Hanxuan 10 × Lumai 14”. ** indicates significant at 0.01 level. LS, low salinity treatment; HS, high salinity treatment

QTLs have been identified for the same or related traits at similar positions [22, 26–29], which confirmed the importance of the loci identified in the present study (Table S7). For example, PH is a crucial agronomic trait for morphogenesis and grain yield formation in wheat [30]. Factors that might influence plant height include Mendelian and quantitative genes that are distributed on all 21 chromosomes [30–33]. More than 20 major genes influencing PH have been identified and designated as *Rht* genes [32, 34–36]. The well-known *Rht4*, *Rht8*, *Rht5*, *Rht-B1*, *RhtD1*, *Rht9*, *Rht12* and *Rht13* genes are located on chromosomes 2BL, 2DS, 3BS, 4BS, 4DS, 5AL, 5AL and 7BS, respectively [14, 37, 38]. During common breeding practices, short genotypes are selected for their lodging resistance. However, in the present study, PH had a consistent positive correlation with YPP under LS and HS conditions, which corroborated similar results obtained under different stress-related environments [39–41]. Here, locus *QPh-4D* was identified on chromosome 4D at 16.93–19.29 Mb under HS conditions and was located in approximately the same region as *RhtD1* based on a comparison with the physical positions of QTLs detected in previous studies [27, 30, 42, 43]. In addition, QTL mapping results of HL-DH population also validated that locus *QPh-4D* was located in a QTL interval (AX-89703298–AX-110564616) affecting PH under LS conditions (Fig. 3a, b) explaining 25.90% of the phenotypic variation (Table 3). The positive additive effect was from Hanxuan10, and lines with the favorable allele GG in AX-89421921 also showed higher PHs in the DH population, which was consistent with the GWAS results (Table 3, Fig. 3c, d). Another locus, *QPh-5A1*, was located on chromosome 5A at 535.91–536.80 Mb under LS and HS treatments. Díaz et al. [44] mapped a QTL (485.83 Mb) affecting PH and SL in the interval *Xmwg522*–*Xcdo412c* on chromosome 5A by analyzing the ITMI wheat mapping population’s response to salinity stress. But this QTL was far away from *QPh-5A1*. Based on the genome sequence from cultivar Chinese Spring (IWGSC V1.1), nine interesting genes were found in the position interval 535.91–536.80 Mb. Gene predication and functional annotation indicated that these genes were related to disease resistance genes and U-box E3 ubiquitin ligase. During the past years, several QTLs conferring disease resistance have been detected in wheat, and studies indicated that these resistance genes not only had effects on disease severity, but also affected growth and yield of wheat [45]. For example, Zheng et al. [46] mapped a large-effect QTL conferring *Fusarium* crown rot resistance in hexaploid wheat and found that the QTL had significant effects on plant height and heading date. While, reports suggested that the U-box E3 ligases played important roles in abiotic stress response. For instance, Min et al. [47] suggested that *CaPUB1*, a hot pepper U-box E3 ubiquitin ligase could enhance the cold tolerance of transgenic rice.

**Table 3:** QTL intervals and agronomic traits revealed by QTL mapping in HL-DH and ZX-RIL populations

Population	Trait	Chromosome	Position (cM)	Left marker	Right marker	LOD	PVE	Add	LeftCI	RightCI
HL-DH	PH-LS	4D	18.8	AX-89421921	AX-110564616	64.35	25.90	6.87	17.45	20.50
ZX-RIL	SPS-HS	5B	92	AX-109996563	AX-111538681	5.03	5.23	− 0.18	91.50	92.50
ZX-RIL	SL-HS	7A	11	AX-108843150	AX-109347413	3.54	3.56	−0.17	10.50	11.50

Position (cM), distance between QTL and the top marker of each linkage map; LOD, threshold of 2.5 was set for declaring the presence of QTL; PVE (%), phenotypic variation explained by QTL; Add Positive ‘additive effect’ indicated an increasing effect from Hanxuan 10 in the HL-DH population, and Zhongmai 175 in ZX-RIL population; negative ‘additive effect’ indicated an increasing effect from Lumai 14 in HL-DH population, and Xiaoyan 60 in ZX-RIL population

*PH* plant height, *SPS* spikelets per spike, *SL* spike length, *HL-DH* the DH population of “Hanxuan 10×Lumai 14”, *ZX-RIL* the RIL population of “Zhongmai 175×Xiaoyan 60”, *LS* low salinity treatment, *HS* high salinity treatment

In this study, TKW value was relatively stable under both LS and HS conditions. Locus *QTkw-4A*, which was significantly associated with TKW under LS conditions, was identified on chromosome 4A at 14.68–16.28 Mb. A common QTL (*Xgwm160*) (719.26 Mb) controlling yield and TKW was detected in a population of 96 DH lines under nutrient-, drought- and salt-stress conditions [39]. A classical grain weight-related gene *TaCWI* had been mapped on chromosomes 4A (610.04 Mb) and 5D (557.95 Mb) in wheat [48]. These QTLs were all different from *QTkw-4A*, implying that *QTkw-4A* could be a novel QTL for TKW. Gene predication and functional annotation of *QTkw-4A* indicated that 15 interesting genes were located in the position interval 14.68–16.28 Mb. Furthermore, one candidate gene encoded a glycerol-3-phosphate acyltransferase (*GPAT*; E.C. 2.3.1.15) was found. The *GPAT* gene has been cloned in some plant, such as *Suaeda salsa*, *Oryza sativa*, tomato, *Arabidopsis*, and so on [49–51]. Studies indicated that the *GPAT* genes were closely related to stress tolerance and seed development [49–51]. Ariizumi et al. [52] found that the content of unsaturated fatty acids increased when overexpressing the *AtGPAT* in rice, which could improve the rates of photosynthesis and growth of transgenic rice seedlings under low temperatures.

SL, a stable phenotypic character, showed significant positive correlations with YPP in two salt-stress environments. In this study, loci *QSl-1B* and *QSl-3B* were identified on chromosome 1B at 674.96–675.13 Mb and on 3B at 62.13–62.14 Mb, respectively. Using the 90 K wheat SNP array, Gao et al. [27] also mapped SNPs on chromosome 1B (*BS00070878_51* – *Kukri_c1529_462*, 22.70 Mb) and 3B (*Ku_c12191_1202—Excalibur_c3556_520*, 815.48 Mb) associated with SL using a Zhou 8425B/Chinese Spring RIL population. Additionally, Mwadzingeni et al. [22] identified markers associated with SL that were located on chromosome 1B using 93 bread wheat genotypes under drought-stress conditions. Liu et al. [53] integrated SL-related QTLs on chromosomes 2A, 2B and 6A on a physical map that was constructed using 192 common wheat samples based on the 90 K SNP chip. The loci *QSl-1B* and *QSl-3B* for SL detected in this study were different from those previous studies and might be novel QTLs. Gene predication and functional annotation indicated that six and two candidate genes were found in the position intervals of *QSl-1B* and *QSl-3B*. These genes were related to keratin-associated protein and ricin B-like lectin gene, respectively. Previous studies suggested that the QTL (*qPE*_*9–1*_) for panicle encoded a protein homologous to the keratin-associated protein 5–4 family in rice [54–56]. While, lectin gene is involved in the abiotic stress response of plants [57]. For instance, the expression level of wheat lectin gene *Hfr-1* was up-regulated under drought conditions, while the other lectin gene *Wci-1* was significantly up-regulated under various stresses [57].

### QTL clusters for grain yield and related traits under salinity stress

Grain yield is a complex quantitative trait and is controlled by multiple genes. Selection based on grain yield alone is simple but ineffective for wheat breeding [58]. Consequently, it is necessary for wheat breeder to identify characteristics that contribute to grain yield [41]. Thus, traits correlated with grain yield will be automatically considered as selective criteria in wheat breeding under any conditions [40, 41]. In this study, BM, SL, GN and SN showed similar and extremely higher correlations with YPP under both LS and HS conditions (Table 2). Which was supported by Mwadzingeni et al. [22] who reported similar correlations between YPP and both BM and GN under drought-stress conditions. QTL clusters and co-localized QTLs for grain yield and component traits related to salinity stress have been reported [26, 39, 59–61]. In addition, an unusually high correlation between agronomic traits controlled by pleiotropic QTLs exists [22, 26, 60–64]. Two occurrences might be responsible for the co-localization of QTL associated with different traits: first, genes affecting various traits or the same traits were strongly linked; and second, single gene might have a series of effects on related traits or affect two or more independent traits [62]. Here, five loci affecting various grain yield-correlated traits were detected on 5A, 5B and 7A under salt-stress conditions (Tables S6).

The group-5 chromosomes appear to harbor genes associated with abiotic stress resistance, and several QTLs for grain yield and related traits under salt-stress conditions were especially located on the group-5 chromosomes of wheat [26, 28, 39, 60, 62]. For example, the gene *Vrn-A1* (587.41 Mb), *Vrn2-5A/ZCCT1-5A* (698.180559 Mb), *Vrn-B1* (573.82 Mb) and *Vrn-D1* (467.18 Mb) that influences grain yield by controlling the vernalization and timing of ear emergence are located on the long arms of chromosome 5A, 5B and 5D [33, 65, 66]. In the present study, locus *Q-5A2* located on chromosome 5A at 692.16–692.39 Mb was identified as affecting YPP and BM under HS conditions. Three loci on chromosome 5B, *Q-5B1* (574.06–578.35 Mb), *QSl-5B2* (583.48–583.89 Mb) and *Q-5B3* (690.08–690.70 Mb), contained SNP clusters associated with YPP, SL, SN, GN and BM under LS conditions. Blast search on the IWGSC database revealed that the locus *Q-5A2* affecting YPP and BM was close to *Vrn2-5A/ZCCT1-5A* (698.180559 Mb) [66], also the locus *Q-5B1* associating with YPP, SL, GN and BM seemed to be located near to the *Vrn-B1* [67]. More importantly, QTL mapping results for the ZX-RIL population validated that locus *Q-5B1* was located in a QTL interval (AX-109996563–AX-111538681) affecting spikelets per spike under HS conditions (Table 3, Fig. S3). In addition, the locus *Q-5B3* approached the QTL (*QKNS.caas-5BL*, 695.99 Mb) of kernel number per spike identified by Li et al. [68]. Results indicated that the co-localized QTL identified here should play a critical role in the yield and its related traits of wheat cultivars grown under stress conditions.

Gene predication and functional annotation of *Q-5A2*, *Q-5B1*, *QSl-5B2* and *Q-5B3* indicated that there were five, 45, 19 and 12 candidate genes contained in the position intervals of each locus, respectively. These genes were related to lipid transfer protein, phytochelatin synthase, serine/threonine protein kinase and MADA-box genes. Lipid transfer proteins (LTPs) are a class of small and ubiquitous proteins and are believed to have critical roles in plant response [69, 70]. Kirubakaran et al. [70] suggested that *Ltp* 3F1 gene from wheat could enhance resistance against fungi in tobacco. Phytochelatin synthases (PCs) are a family of cysteine-rich thiol-reactive and heavy metal-binding peptides involved in heavy metal accumulation and detoxification of plants [71]. Studies indicated that the heteroexpression of wheat *TaPCS1* enhanced the Cd accumulation in rice shoots [71]. Serine/threonine protein kinase (STK) is a large protein family which has an important role in stress signaling and responses of plants [72]. Ge et al. [73] demonstrated that the excess-expression of *TaSTK* gene was capable of increasing the root growth of *Arabidopsis thaliana* under salt stress. Besides, the MADS-box family members encode transcription factors participating not only in floral organ specification, but also in plant growth and development [74]. Ma et al. [75] characterized 180 MADS-box genes in wheat and found them involved in both abiotic and biotic stress responses.

**Fig. 4:**
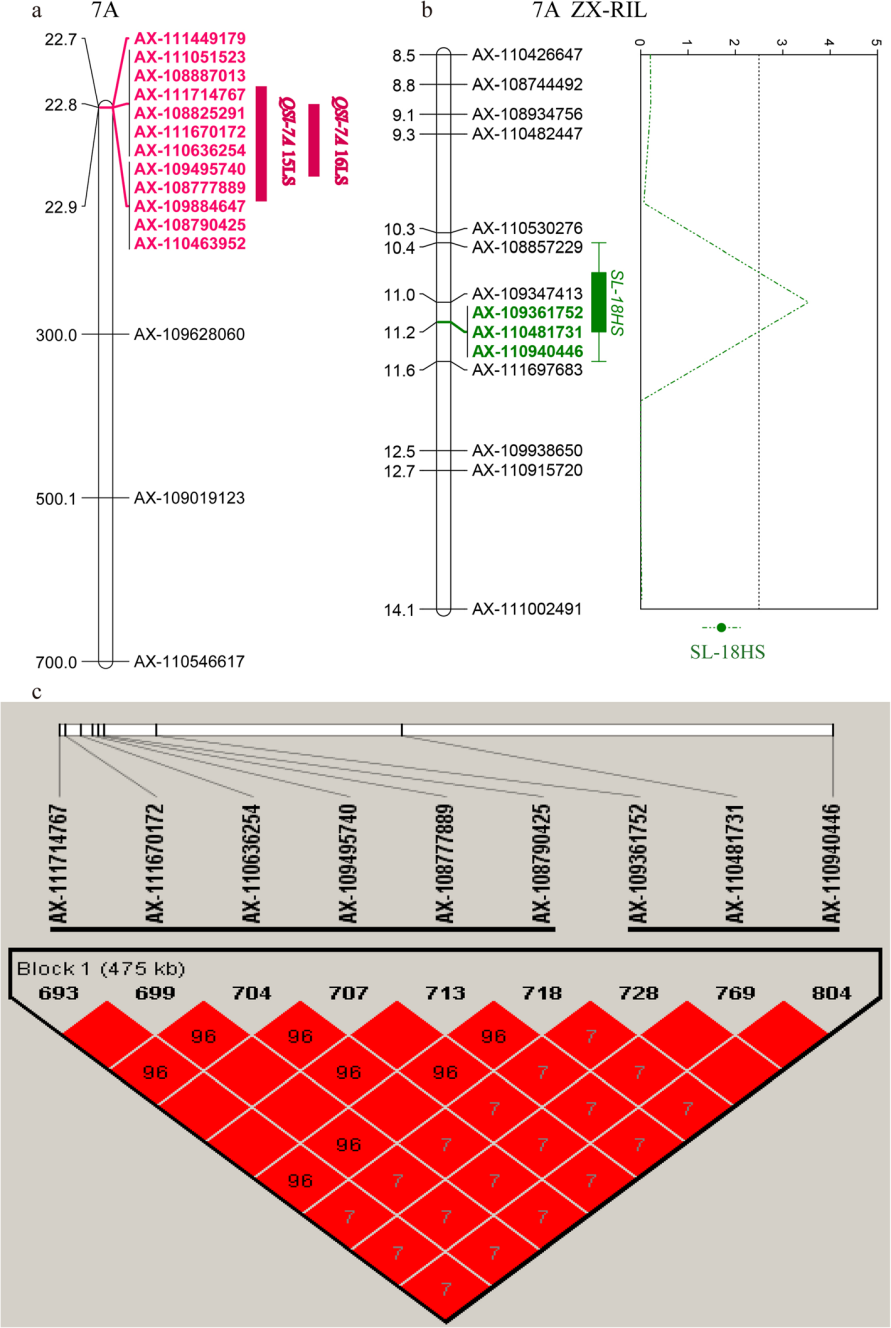
QTL position on chromosome 7A for spike length (SL) in the common wheat population and ZX-RIL population. **a** Chromosome location of *QSl-7A* in the 191 common wheat accessions. **b** QTL position for SL in RIL population of “Zhongmai 175×Xiaoyan 60”. **c** LD plot of the *QSl-7A* in the 191 common wheat accessions and the QTL for SL in RIL population of “Zhongmai 175 × Xiaoyan 60”

**Fig. 5:**
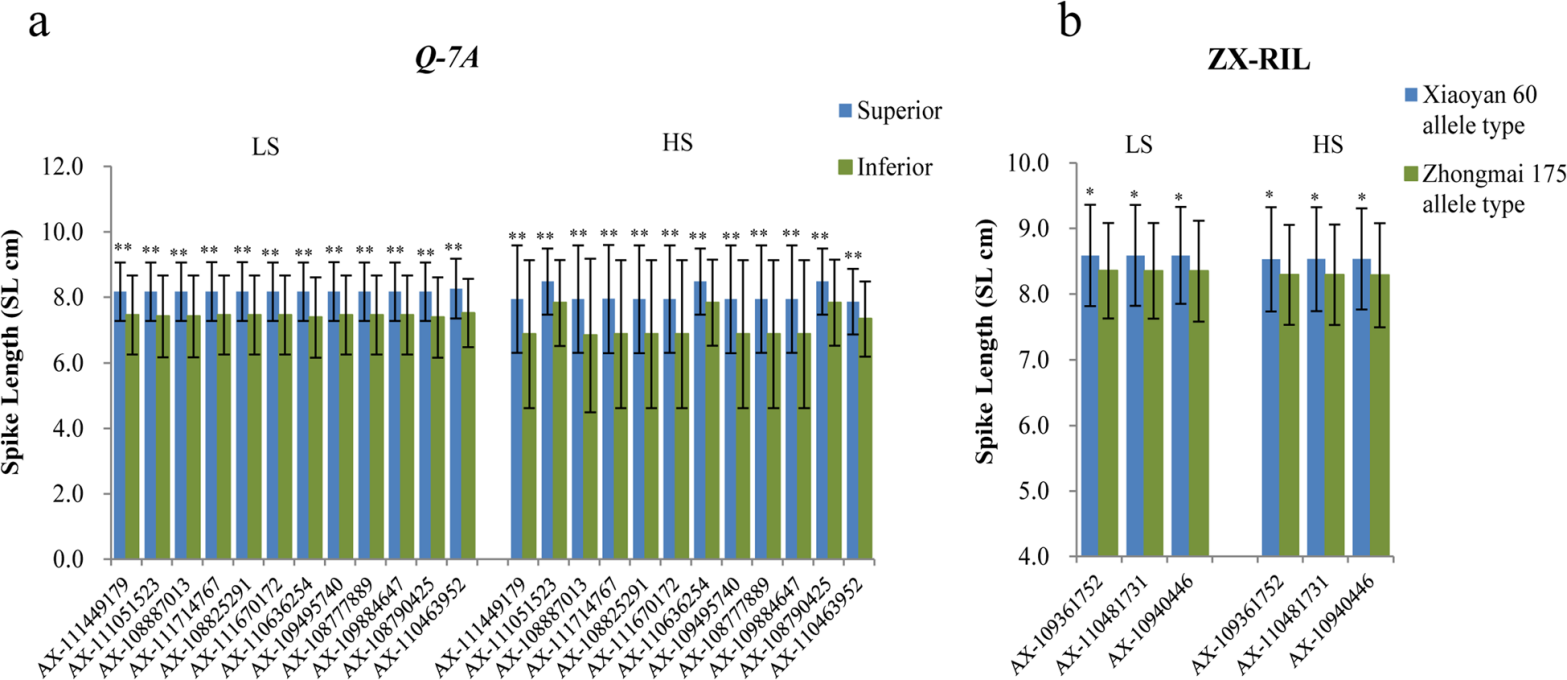
The spike length of accessions with superior allele of *Q-7A* in the common wheat population and ZX-RIL population. **a** The spike length of accessions with superior allele of *Q-7A* in the common wheat population. **b** The spike length of accessions with superior allele of QTL for SL in RIL population of “Zhongmai 175×Xiaoyan 60”. ** and * indicate significant at 0.01 and 0.05 levels, respectively. LS, low salinity treatment; HS, high salinity treatment

Additionally, the locus effecting YPP was located on chromosome 7A (*Q-7A*, 22.75–22.86 Mb) and also contained a SNP cluster associated with SL under salt-stress conditions. On chromosome 7A, Gao et al. [27] identified QTL (*wsnp_Ex_c200_391493* – *Ex_c52798_415*, 244.27 Mb) for TKW, SN, SL and PH. Additionally, QTL mapping results in the ZX-RIL population verified that locus *Q-7A* could form a LD block (475 kb) with the QTL interval (AX-108843150–AX-109347413) that affected SL under HS treatments (Fig. 4a–c). The positive additive effect was from Xiaoyan 60, and cultivars with favorable alleles also showed longer SLs in the association population, which was consistent with the GWAS results (Table 3, Fig. 5a, b).

### Favorable haplotypes for wheat salt-tolerance breeding

Using the base types of SNP markers and distributions of alleles associated with a trait, some haplotypes were identified, and favorable haplotypes were determined by detecting their phenotypic values using *t*-tests. After removing the haplotypes less than 5%, the main haplotypes of each locus were used for further analysis. The *t*-tests indicated that cultivars with favorable haplotypes usually had more desirable phenotypes under salt-stress conditions (Table 4, Fig. S5). In addition, the proportion of favorable haplotype for each locus was various, which indicated that these important loci had experienced different degrees of selection during wheat breeding. For example, the proportions of favorable haplotypes for locus *QYpp-3A* and *QTkw-4A* were only 5.24 and 8.90%, respectively, which implied that these loci had not experienced strong selection.

**Table 4:** Haplotype analysis of the important loci

Locus	Traits	No. of SNPs	No. of haplotypes	Sequence of alleles	Frequency (%)	Traits	15LS Mean ± S.D	16LS Mean ± S.D	17LS Mean ± S.D	15HS Mean ± S.D	16HS Mean ± S.D	17HS Mean ± S.D
*QSl-1B*	SL	11	2	TCTCATCCCCT**	33.51	SL	9.65 ± 0.99	7.77 ± 1.01	8.54 ± 0.85	8.57 ± 1.17	7.31 ± 1.24	8.89 ± 0.98
				CTCTGAGATTC	52.88	SL	8.95 ± 1.09	7.05 ± 0.96	7.87 ± 0.67	7.79 ± 1.01	6.71 ± 1.02	8.20 ± 0.90
*QYpp-3A*	YPP	6	3	CCAATG**	5.24	YPP	26.56 ± 4.85	22.57 ± 5.17	24.84 ± 3.63	14.36 ± 1.94	19.37 ± 3.89	23.21 ± 5.17
				ATAATG	77.49	YPP	19.33 ± 6.49	18.74 ± 5.24	19.25 ± 4.20	10.43 ± 3.67	12.96 ± 5.45	16.61 ± 4.95
				ATGGGA	4.71	YPP	17.65 ± 7.75	16.13 ± 5.59	18.31 ± 4.84	9.22 ± 4.24	10.02 ± 5.51	17.60 ± 4.04
*QSl-3B*	SL	5	2	CAGTT**	30.37	SL	9.38 ± 1.50	7.70 ± 1.02	–	8.36 ± 1.48	–	–
				TGTCA	65.97	SL	8.84 ± 1.77	7.16 ± 0.91	–	7.60 ± 1.75	–	–
*QTkw-4A*	TKW	25	2	CCGCCCGGGCGTGCGCGCTGGATCC**	8.90	TKW	–	52.36 ± 5.43	46.03 ± 4.75	–	49.14 ± 5.77	40.20 ± 4.80
				TTATTGATATACCTATATCCTGAGT	83.77	TKW	–	47.92 ± 6.02	40.92 ± 4.79	–	43.60 ± 9.83	37.65 ± 4.99
*QPh-4D*	PH	4	2	GCTG**	68.06	PH	78.14 ± 20.67	77.55 ± 13.49	82.70 ± 14.71	63.40 ± 17.55	58.83 ± 15.80	73.26 ± 12.67
				TTCA	28.27	PH	67.92 ± 6.00	65.98 ± 5.81	69.37 ± 6.02	57.58 ± 4.86	53.96 ± 6.53	60.63 ± 6.47
*Q-5A2*	BM YPP	4	2	GACC**	58.64	BM	–	–	–	22.84 ± 6.53	–	–
				AGAT	37.17	BM	–	–	–	19.93 ± 8.86	–	–
				GACC**	58.64	YPP	–	–	–	11.35 ± 3.11	–	–
				AGAT	37.17	YPP	–	–	–	9.73 ± 4.30	–	–
*Q-5B1*	SL BM GN YPP	4	2	AGCA**	88.48	BM	–	–	–	22.46 ± 8.29	26.54 ± 10.51	–
				GTTG	4.71	BM	–	–	–	15.55 ± 5.51	9.04 ± 6.53	–
				AGCA**	88.48	SL	–	–	–	–	6.94 ± 1.22	–
				GTTG	4.71	SL	–	–	–	–	5.45 ± 2.54	–
				AGCA**	88.48	YPP	–	19.28 ± 5.59	–	11.11 ± 3.93	13.88 ± 5.48	–
				GTTG	4.71	YPP	–	15.33 ± 4.65	–	7.30 ± 2.45	4.51 ± 3.67	–
*QSl-5B2*	SL	21	2	CTTTTAGCGACCGGGCGGCCC**	85.86	SL	–	–	–	–	6.89 ± 1.35	–
				TCCCCGAGAGTAAAAAATGTA	4.71	SL	–	–	–	–	4.58 ± 2.88	–
*Q-5B3*	SN BM YPP GN	36	2	GCCCCCTGGCTCCGCCACGCCGGGGGCGTCGTGGCG**	54.45	BM	49.02 ± 16.02	–	–	23.90 ± 8.11	–	–
				TAAGGTCAATCATATTGTATTAATAAAACTTCCATT	12.57	BM	35.86 ± 15.96	–	–	18.35 ± 8.87	–	–
				GCCCCCTGGCTCCGCCACGCCGGGGGCGTCGTGGCG**	54.45	GN	627.45 ± 194.97	415.44 ± 107.02	–	301.59 ± 113.55	310.88 ± 116.97	–
				TAAGGTCAATCATATTGTATTAATAAAACTTCCATT	12.57	GN	448.46 ± 203.83	361.03 ± 132.06	–	221.08 ± 105.19	255.05 ± 144.92	–
				GCCCCCTGGCTCCGCCACGCCGGGGGCGTCGTGGCG**	54.45	SN	15.43 ± 4.51	10.69 ± 2.34	–	6.75 ± 2.32	–	–
				TAAGGTCAATCATATTGTATTAATAAAACTTCCATT	12.57	SN	11.31 ± 5.38	9.43 ± 3.04	–	5.33 ± 2.46	–	–
				GCCCCCTGGCTCCGCCACGCCGGGGGCGTCGTGGCG**	54.45	YPP	21.65 ± 6.80	–	–	11.78 ± 3.73	–	–
				TAAGGTCAATCATATTGTATTAATAAAACTTCCATT	12.57	YPP	16.43 ± 7.39	–	–	9.17 ± 4.39	–	–
*Q-7A*	SL YPP	12	2	CGTTACGACCCT**	90.58	SL	–	7.39 ± 1.02	8.18 ± 0.90	–	–	8.49 ± 1.12
				TTACGTAGATAA	5.24	SL	–	6.77 ± 0.87	7.28 ± 1.31	–	–	7.76 ± 1.42
				CGTTACGACCCT**	90.58	YPP	23.71 ± 6.62	–	–	–	–	–
				TTACGTAGATAA	5.24	YPP	19.08 ± 8.93	–	–	–	–	–

*PH* plant height, *SN* spike number, *SL* spike length, *GN* grain number, *TKW* thousand kernels weight, *YPP* yield per plot, *BM* biological mass, *LS* low salinity treatment, *HS* high salinity treatment, *S. D* standard deviation

** represents the favorable haplotype

“--” represents no significantly difference between haplotypes

**Fig. 6:**
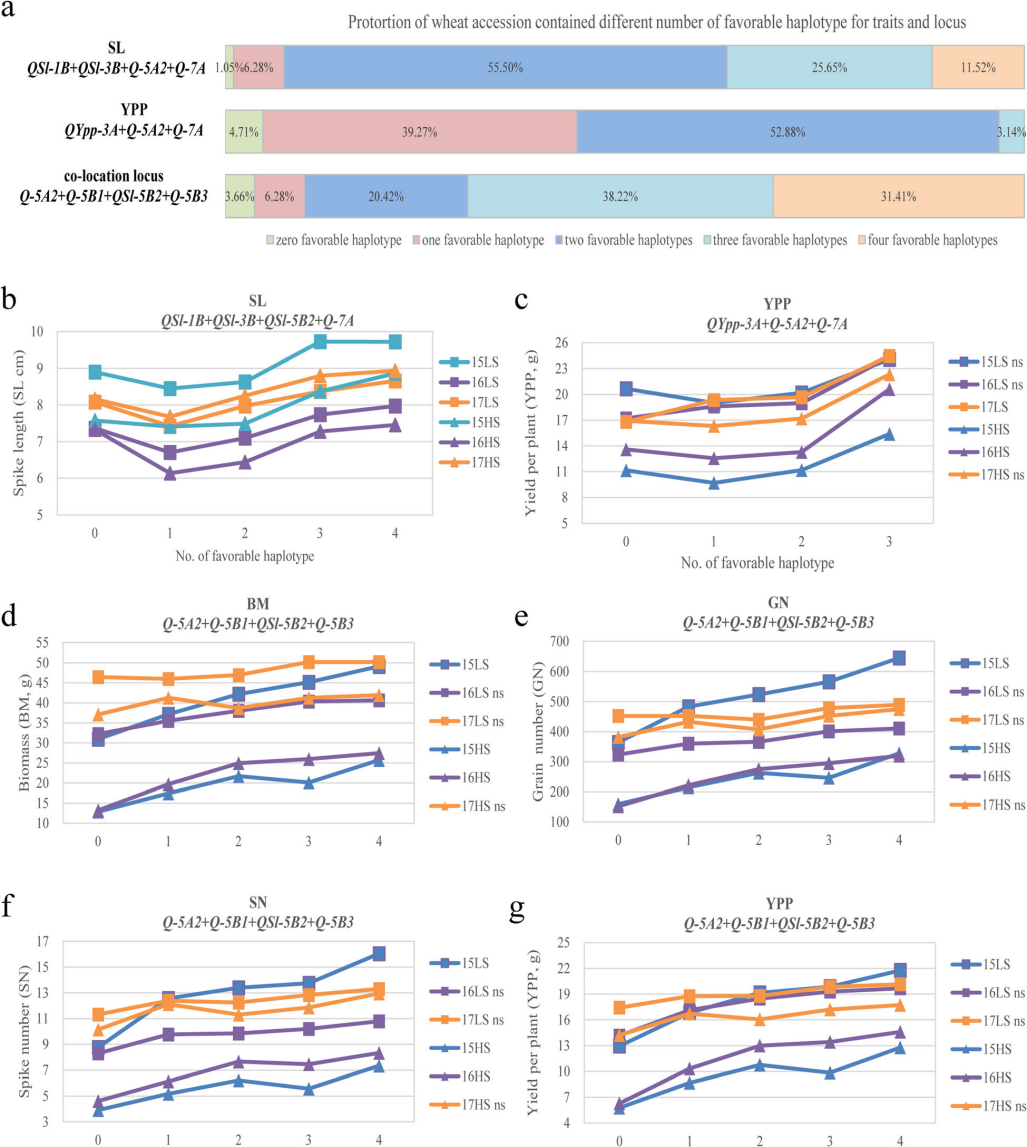
The proportions and phenotype values of wheat accessions contained different number of favorable haplotypes for traits and loci. **a** The proportion of wheat accessions contained different number of favorable haplotype for spike length (SL), yield per plot (YPP) and the co-location loci on the group-5 chromosomes. The percentage represents the proportion of wheat accessions. **b** Spike length comparison of wheat accessions contained different number of favorable haplotypes of loci associated with SL. **c** Yield comparison of wheat accessions contained different number of favorable haplotypes of loci associated with YPP. **b**-**g** Phenotype comparison of wheat accessions contained different number of favorable haplotypes of co-location loci on the group-5 chromosomes. LS, low salinity treatment; HS, high salinity treatment. ns represents no significantly difference

Further comparative analyses of locus effects revealed that cultivars with three or more favorable haplotypes of loci *QSl-1B*, *QSl-3B*, *QSl-5B2* and *Q-7A* had obviously longer SLs under LS and HS conditions (Fig. 6). About 37.17% of the population used in this study, such as landrace Fumai and improved variety Han 4589, carried three or more superior haplotypes for SL, which implied that these loci had aggregated during wheat breeding (Fig. 6). Cultivars with three favorable haplotypes at loci *QYpp-3A*, *Q-5A2*, and *Q-7A* produced greater yields under both salt-stress conditions (Fig. 6). However, only 3.14% of the population, such as some well-known salt-tolerant cultivars Cang 6001 and Cang 6005, contained three haplotypes for yield, demonstrating that these superior haplotypes had not been fully utilized during salt-tolerant wheat breeding (Fig. 6). For the co-localization loci of the group-5 chromosomes, namely *Q-5A2*, *Q-5B1*, *QSl-5B2* and *Q-5B3*, cultivars with four favorable haplotypes had the highest SN, GN, BM and yield values under LS and HS conditions (Fig. 6). Not only some well-known salt-tolerant cultivars, such as Cang 6002, Cang 6005, Dekang 961, Keyi 26 and Kenong 199, but also Xiaoyan derivatives, such as Xiaoyan 6, Xiaoyan 926, Xiaoyan 54 and Xiaoyan 101, possessed four favorable haplotypes of these loci, and approximately 31.41% of cultivars contained four superior haplotypes of these co-localized loci of the group-5 chromosomes (Fig. 6), which indicated that these loci had been integrated during wheat breeding and had potential for increasing wheat salt tolerance. In general, the great differences in the distributions of favorable haplotypes in the population indicated that there were various genetic bases of salt tolerance in different varieties, and the accessions that possessed the superior haplotypes, such as cultivars Cang 6001, Cang 6002, Cang 6005, Jimai 32 and some Xiaoyan derivatives, could play key roles in wheat salt-tolerance breeding.

### Development of molecular markers for marker-assisted selection

Some studies have emphasized that the salt-tolerance of wheat was closely linked with the ability to exclude Na^+^ from leaf [20, 59, 63]. Genc et al. [59] identified five QTLs associating with Na^+^ exclusion under hydroponics-based seedling assays. The two Na^+^ exclusion genes, *Nax1* and *Nax2*, also have been found in durum wheat [76, 77]. However, the main QTLs identified in the present study were not co-located with mapped genes or QTLs for Na^+^ exclusion. Reports suggested that QTLs for Na^+^ exclusion identified in hydroponics had dinky impacts on wheat yield [63, 78] and QTLs related with salt-tolerance generally varied depending on populations and environments [63]. Actually, the QTLs associating with yield and related traits in the salt stress field can play a more important role in the wheat breeding practices. Although, with the availability of next-generation sequencing, a large number of SNPs have recently been discovered in wheat [79]. Different types of high-throughput SNP arrays have been successfully used for studying marker-trait associations in wheat populations [79]. However, the validation and utilization of these markers still represent major challenges owing to the time and labor required for genotyping huge progeny pools [79, 80]. Recently, KASP genotyping technology was developed by LGC limited [81] for SNP genotyping. However, only a few KASP markers have been developed for important agronomic traits in wheat [14]. In this study, 14 KASP markers were successfully developed for nine loci associating with yield and related traits. These KASP markers could accurately classify two alleles in the natural population (Fig. S6). KASP markers related to BM, GN, SL and YPP came from two pleiotropic loci, namely *Q-5A2*, affecting BM and YPP under HS conditions, and *Q-7A*, affecting SL and YPP under LS conditions. These KASP markers will be useful for screening salt-tolerant wheat germplasms and improving the selection efficiency of wheat salt-tolerance breeding.

## Conclusion

In this study, 191 wheat accessions were evaluated in six environments for yield and related traits under low and high salinity and were genotyped using Wheat660K SNP array. GWAS analysis showed that a total of 389 SNPs was significantly associated with eight traits in wheat under two different salinity conditions, and 11 loci could have key roles in affecting plant height, yield and related traits of wheat under salt stress. Four loci with high frequencies of favorable alleles were identified for YPP, SN, SL, GN and BM in position 692.16–692.39 Mb on chromosome 5A and in positions 574.06–578.35 Mb, 580.03–584.89 Mb, and 690.08–690.70 Mb on chromosome 5B. For nine loci that closely linked with yield and related traits, 14 KASP markers were successfully developed and three of them were validated in a bi-parental population derived from Hanxuan 10 and Lumai 14. These findings provided useful information for wheat salt-tolerance improvement by marker assisted selection in future.

## Methods

### Plant materials

The plant material was a collection of 191 wheat accessions including five introduced varieties, ten landraces and 176 improved cultivars (Table S1). Except for one improved cultivar from an unknown source, these genotypes originated from Chile (1), Italy (2), Romania (1), Soviet Union (1) and Chinese provinces including Anhui (1), Beijing (17), Gansu (3), Hebei (40), Henan (30), Inner Mongolia (1), Jilin (1), Qinghai (6), Shaanxi (39), Shandong (34), Shanxi (8), Sichuan (3), Tianjin (1) and Tibet (1). Details for each accession were given in Table S1. The sampling was approved by the Institute of Genetics and Developmental Biology, Chinese Academy of Sciences (Beijing, China). And all the plant materials were provided by the Institute of Genetics and Developmental Biology, Chinese Academy of Sciences (Beijing, China).

Significant SNPs revealed by GWAS were validated by a RIL population Zhongmai 175/Xiaoyan 60 with 350 F_7_ lines (ZX-RIL) and a Double Haploid population Hanxuan 10/ Lumai 14 (HL-DH) with 150 lines.

### Field experiments and phenotyping

The 191 accessions were planted and harvested at Nanpi Eco-Agricultural Experimental Station, Chinese Academy of Sciences (Cangzhou, Hebei, 38°00′N, 116°40′E, Altitude 11 m) in 2014–2015, 2015–2016, and 2016–2017 cropping seasons. This station is located in the warm temperate semi-humid monsoon climate zone, with average annual temperature 12.3 °C and average annual precipitation 480 mm. The normal soil in Nanpi Eco-Agricultural Experimental Station is typical in drought and salt stress around Bohai Sea region and represents LS treatment. The salinity of normal soil in Nanpi was 0.18% (m/m) according to the detection values in decades. HS treatment was set in salt pools which were artificially made. The soil salinity of salt pools was guaranteed at 0.3% (m/m) by adding sea-salt solution with specific concentration into normal soil. The total salt content of the soil was determined by chloride-Silver nitrate titration method. Hence, two treatments, LS (0.18%, m/m) and HS (0.3%, m/m) were set. Totally, six salinity environments were designated as 15LS, 15HS, 16LS, 16HS, 17LS and 17HS. Each salt pool was a cement pond of 6.6 m × 2 m × 1.5 m, with waterproof cement thickness of 10 cm at the bottom. The bottom of the cement pond was paved with stones and river sand, and covered with non-woven fabrics. The soil evenly mixed with sea-salt solution was filled in the whole cement pond. The salt concentration of salt pool was checked from time to time, with the assurance that the salinity was maintained at 0.3% (m/m). The LS and HS treatments did not involve artificial irrigation during the cropping seasons. The field experiments were performed using a completely randomized design with five replications. Each block contained sixteen 200-cm-long rows with 10 cm between neighboring plants and 23 cm between neighboring rows. Hole sowing method was used and three sprouted seeds of any accession were sown in each plot. The three plants in one hole were harvest together and eight phenotypic traits at adult stage were investigated, including plant height (PH), spike number (SN), spike length (SL), kernels per spike (KPS), grain number (GN), thousand kernel weight (TKW), yield per plot (YPP) and biological mass (BM). Measurement methods for each trait were as follows: PH was the distance between the stem base and the top of the main spike excluding awns, SN was the number of spikes per plot, SL was the length of the main spike per plot, KPS was the kernel number of main spike per plot, GN was the number of grains per plot, TKW was the calculated by (YPP/GN) × 1000, YPP was the weight of grains per plot, BM was the aboveground weight per plot. The relative value of high salinity/low salinity (HS/LS) for each trait was also calculated and used for genome-wide association analysis.

The phenotypes of ZX-RIL and HL-DH were used to validate significant SNPs detected by GWAS. These two populations were planted and harvested under LS and HS conditions at Nanpi Eco-Agricultural Experimental Station in 2016–2017 (ZX-RIL) and 2017–2018 (ZX-RIL and HL-DH). Three repeated rows and ten replicated individuals were set for low and high salinity treatment, respectively. Seeds were sowed with row space 20 cm and plant space 10 cm. Phenotypic traits were investigated as mentioned above.

### Genotyping

Genomic DNA was extracted from fresh leaves collected from the 191 accessions using a CTAB method [82] . Then, DNA samples were sent to the Capital Bio® genotyping facility (Beijing, China) for SNP genotyping with the Axiom Wheat660K Genotyping Array (https://wheat.pw.usda.gov/ggpages/topics/Wheat660_SNP_array_developed_by_CAAS.pdf) according to the manufacturer’s instructions (Axiom 2.0 Assay Manual Workflow User Guide Rev3). Markers were removed from the dataset for SNP call rate less than 80% and minor allele frequencies less than 0.05. This quality preprocessing of genotyping data was completed using PLINK software (http://pngu.mgh.harvard.edu/purcell/plink/) [83].

### Statistical analysis

#### Phenotypes

Phenotypic data were analyzed with SAS v.9.2 software (SAS Institute Inc., Cary, NC). Analyses of variance (ANOVA), co-efficient of correlation and repeatability were calculated for all traits. Heritability estimates were calculated using the formula: *H*^2^ = σ^2^_g_/ (σ^2^_g_ + σ^2^_ge_/*r* + σ^2^_ε_/*re*), where σ^2^_g_, σ^2^_ge_ and σ^2^_ε_ were estimates of genotype, genotype × environment interaction and residual error variances, respectively, and e and r represented the number of environment and replicates, respectively.

#### Population structure and kinship analysis

Linkage disequilibrium (LD) among markers was investigated using TASSEL 5.0 with the markers whose positions were known [84]. A distance matrix was established by the SNPs shared by all the accessions using PLINK [83]. Based on this distance matrix, a neighbor-joining tree was constructed using PHYLIP software. Kinship matrixes (the K model) were established to infer relationship among accessions using TASSEL 5.0 [84]. Population structure (the Q matrix) was calculated using the Bayesian clustering program Structure V2.3.4 [85]. The structure program was run ten times for each subpopulation (k) value, ranging from 1 to 15, with a burn-in period at 100,000 iteration and a run of 500,000 replication of Markov Chain Monte Carlo (MCMC) after burn in [85]. The subpopulation number was estimated using theΔK method [86]. Meanwhile, Principal Component Analysis was performed to infer population stratification via TASSEL 5.0 [84].

#### Genome-wide association analysis

Associations between SNPs and phenotypic variations were tested with mixed linear model (MLM) by TASSEL 5.0 [84]. And two factors including population structure (Q matrix) and relationship kinship (K model) were used as confounding factors [87, 88]. The first five principal components of SNPs were leaded in the GWAS model. A GAPIT package [89] in R software using MLM was conducted to check the consistency and accuracy of the results from TASSEL.

#### Development of KASP markers for association SNPs

The Kompetitive Allele Specifc Polymerase Chain Reaction (KASP) (LGC Genomics LLC, Beverly, MA, USA) markers were developed to detect and distinguish alleles of key SNPs for the important loci. One or two representative SNPs were selected for each locus, excepting for the loci of PH. A total of 14 SNPs were converted to KASP markers for nine loci. Sequences from the Wheat660K array flanking the SNP were used for KASP primer design by Primer Premier 5 software. KASP reactions and detection of the fluorescence signal were performed according to the KASP manual by Capital Bio® genotyping facility (Beijing, China).

## Supplementary Information

**Additional file 1: Table S1.** Name and origin of the 191 common wheat accessions used in this study.

**Additional file 2: Table S2.** Correlation analysis of the field experiments from 2015 to 2017 under low and high salinity treatments. PH, plant height; SN, spike number; SL, spike length; KPS, kernels per spike; GN, grain number; TKW, thousand kernels weight; YPP, yield per plot; BM, biological mass. ** represented significant difference determined at *p*<0.01. LS, low salinity treatment; HS, high salinity treatment.

**Additional file 3: Table S3.** Genetic diversity in each chromosome was calculated according to genotypic data of Wheat660K SNPs chip in the 191 wheat accessions under low and high salinity treatments.

**Additional file 4: Table S4.** GWAS results of the eight agronomic traits among the 191 common wheat accessions under low and high salinity treatments. PH, plant height; SN, spike number; SL, spike length; KPS, kernels per spike; GN, grain number; TKW, thousand kernels weight; YPP, yield per plot; BM, biological mass. LS, low salinity treatment; HS, high salinity treatment; UN, unknown.

**Additional file 5: Table S5.** Candidate SNPs revealed by GWAS in 191 common wheat accessions under low and high salinity treatments. PH, plant height; SN, spike number; SL, spike length; KPS, kernels per spike; GN, grain number; TKW, thousand kernels weight; YPP, yield per plot; BM, biological mass. LS, low salinity treatment; HS, high salinity treatment.

**Additional file 6: Table S6.** Favored alleles and genetic effects of 11 loci associated with traits under low and high salinity treatments. PH, plant height; SN, spike number; SL, spike length; KPS, kernels per spike; GN, grain number; TKW, thousand kernels weight; YPP, yield per plot; BM, biological mass. LS, low salinity treatment; HS, high salinity treatment. “ns” represents no calculation for no significantly difference of traits between alleles.

**Additional file 7: Table S7.** SNPs loci significantly associated with yield-related traits identified in the present and previous studies. PH, plant height; SN, spike number; SL, spike length; KPS, kernels per spike; GN, grain number; TKW, thousand kernels weight; YPP, yield per plot; BM, biological mass.

**Additional file 8: Table S8.** The information of the 14 KASP markers developed for nine loci. PH, plant height; SN, spike number; SL, spike length; KPS, kernels per spike; GN, grain number; TKW, thousand kernels weight; YPP, yield per plot; BM, biological mass.

**Additional file 9: Figure S1.** The average number of favorable alleles of common wheat accessions. (a) The average number of favorable alleles of common wheat accessions from different types. (b) The average number of favorable alleles of common wheat accessions from different regions.

**Additional file 10: Figure S2.** Mean difference in different traits between HX and LM alleles in DH population of “Hanxuan 10×Lumai 14”, where HX indicated the “Huanxuan 10” allele and LM indicated “Lumai 14” allele (** indicates significant at 0.01 level).

**Additional file 11: Figure S3.** QTL position on chromosome 5B for spike length (SL) in the common wheat population and ZX-RIL population. (a) Chromosome location of *Q-5B1* in the 191 common wheat accessions. (b) QTL position for SPS in RIL population of “Zhongmai 175×Xiaoyan 60”. (c) LD plot of the *QSl-5B1* in the 191 common wheat accessions and the QTL for SPS in RIL population of “Zhongmai 175 × Xiaoyan 60”.

**Additional file 12: Figure S4.** The proportion of different haplotypes for each locus. The percentage represented the proportion of haplotype in the 191 common wheat accessions.

**Additional file 13: Figure S5.** Haplotype analysis of the important loci. PH, plant height; SN, spike number; SL, spike length; GN, grain number; TKW, thousand kernels weight; YPP, yield per plot; BM, biological mass. LS, low salinity treatment; HS, high salinity treatment.

**Additional file 14: Figure S6.** Scatter plots of the 14 KASP markers for nine key loci identified in the present study. Red dots indicate the accessions had the FAM-type allele; blue dots indicate the accessions had the HEX-type allele.

## Data Availability

The data sets supporting the results of this study are included within the article and its additional files. The experiment data reported in this study have been deposited in the Genomic Expression Archive (GEA) in Bioinformation and DDBJ Center under accession number E-GEAD-406 at https://ddbj.nig.ac.jp/gea/reviewer, the access key is “bDhkCrXEb17jzYrxoMpt”. The genotype data have been also deposited in the Genome Variation Map (GVM) in Big Data Center, Beijing Institute of Genomics (BIG), Chinese Academy of Science, under accession numbers GVM000089 at http://bigd.big.ac.cn/gvm/getProjectDetail?project=GVM000089.

## Abbreviations

BM: Biological mass
GN: Grain number
GWAS: Genome-wide association studies
KPS: Kernels per spike
PH: Plant height
QTL: Quantitative trait loci
SN: Spike number
SNP: Single nucleotide polymorphism
SL: Spike length
TKW: Thousand kernels weight
YPP: Yield per plot
