# Supplementary figures and images for: Genome-wide association study of yield and related traits in common wheat under salt-stress conditions
Source: BMC Plant Biol. 2021 Jan 7;21:27. doi: 10.1186/s12870-020-02799-1 (PMC7792188; doi:10.1186/s12870-020-02799-1)

**a**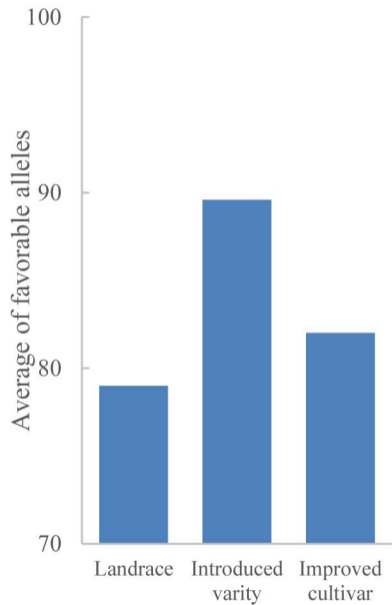**b**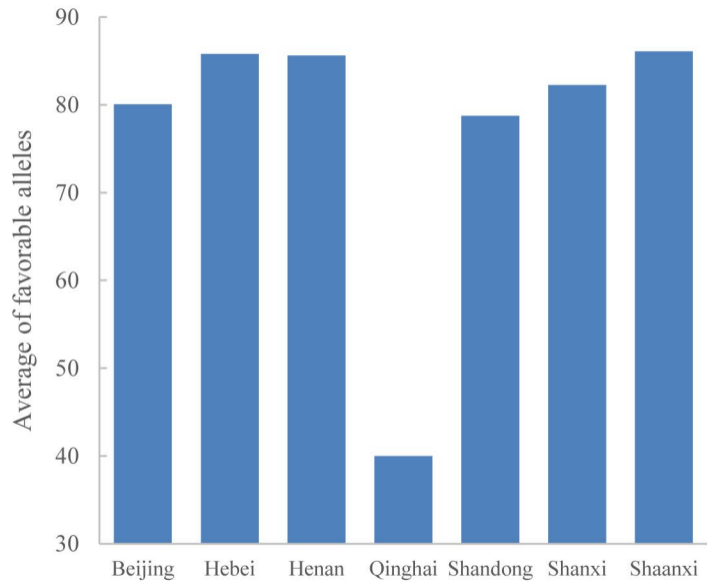

Supplement: Supplementary file 9 — Additional file 9: Figure S1. The average number of favorable alleles of common wheat accessions. (a) The average number of favorable alleles of common wheat accessions from different types. (b) The average number of favorable alleles of common wheat accessions from different regions. [file 12870_2020_2799_MOESM9_ESM.pdf]

a

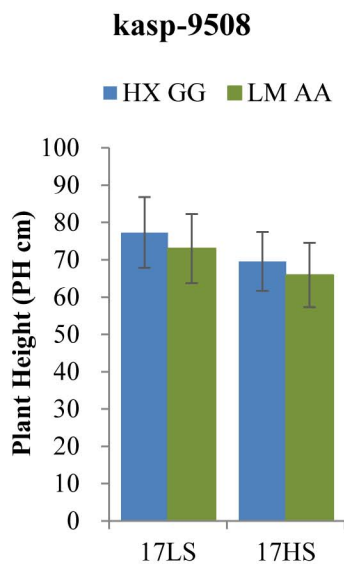

b

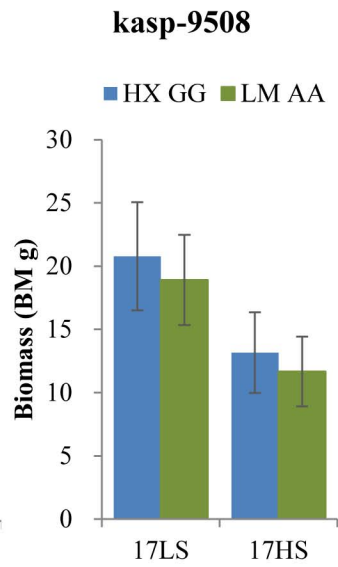

c

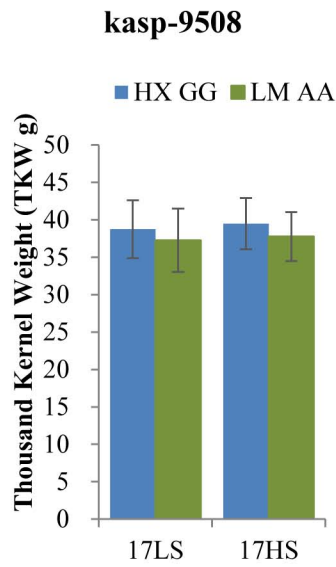

d

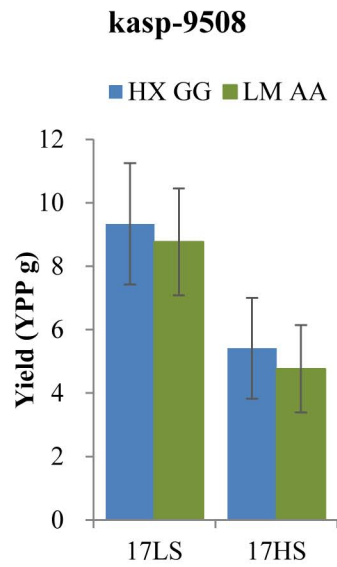

e

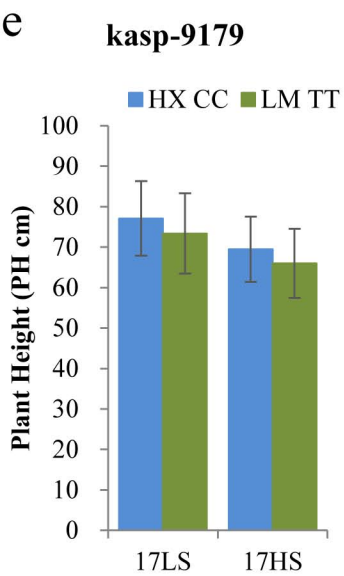

f

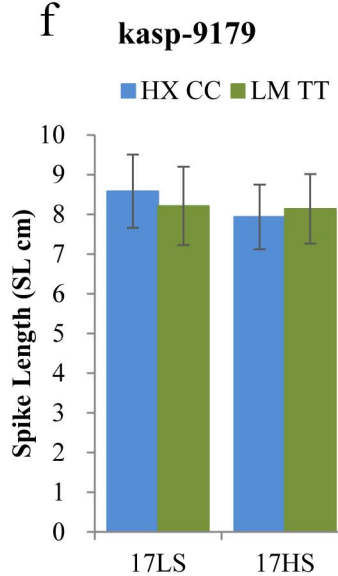

g

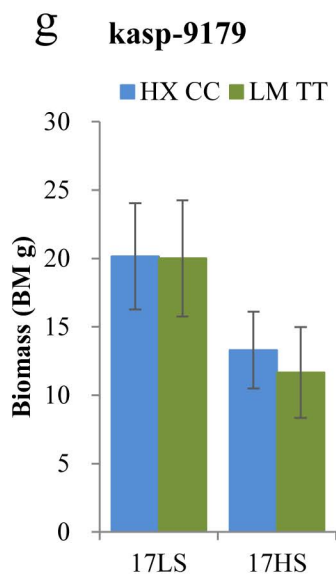

h

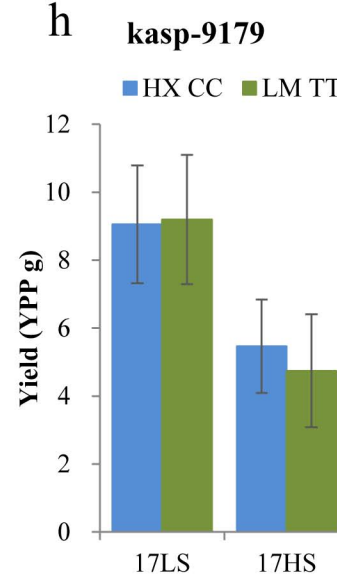

i

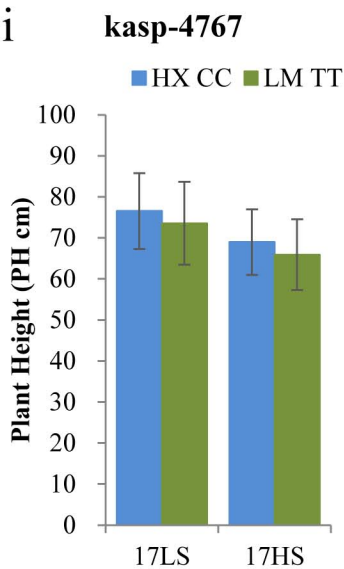

j

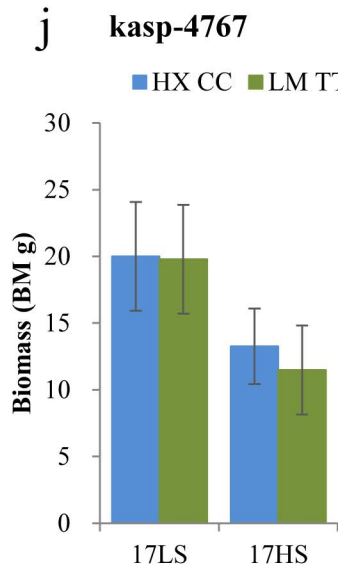

k

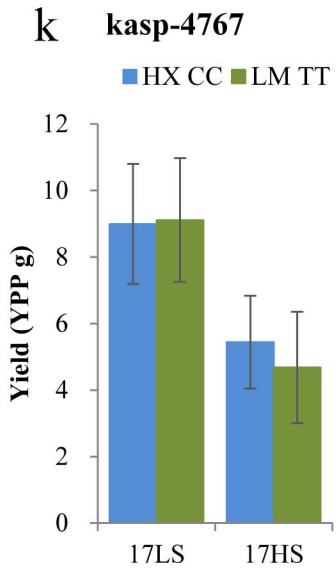

l

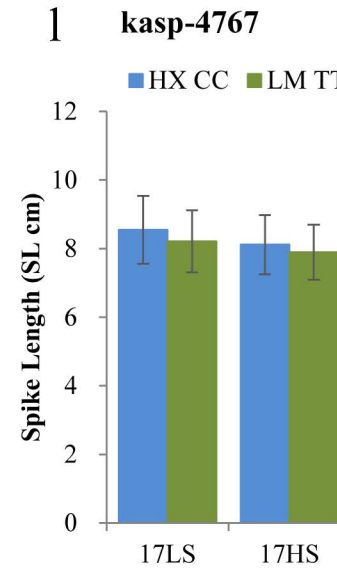

Supplement: Supplementary file 10 — Additional file 10: Figure S2. Mean difference in different traits between HX and LM alleles in DH population of “Hanxuan 10×Lumai 14”, where HX indicated the “Huanxuan 10” allele and LM indicated “Lumai 14” allele (** indicates significant at 0.01 level). [file 12870_2020_2799_MOESM10_ESM.pdf]

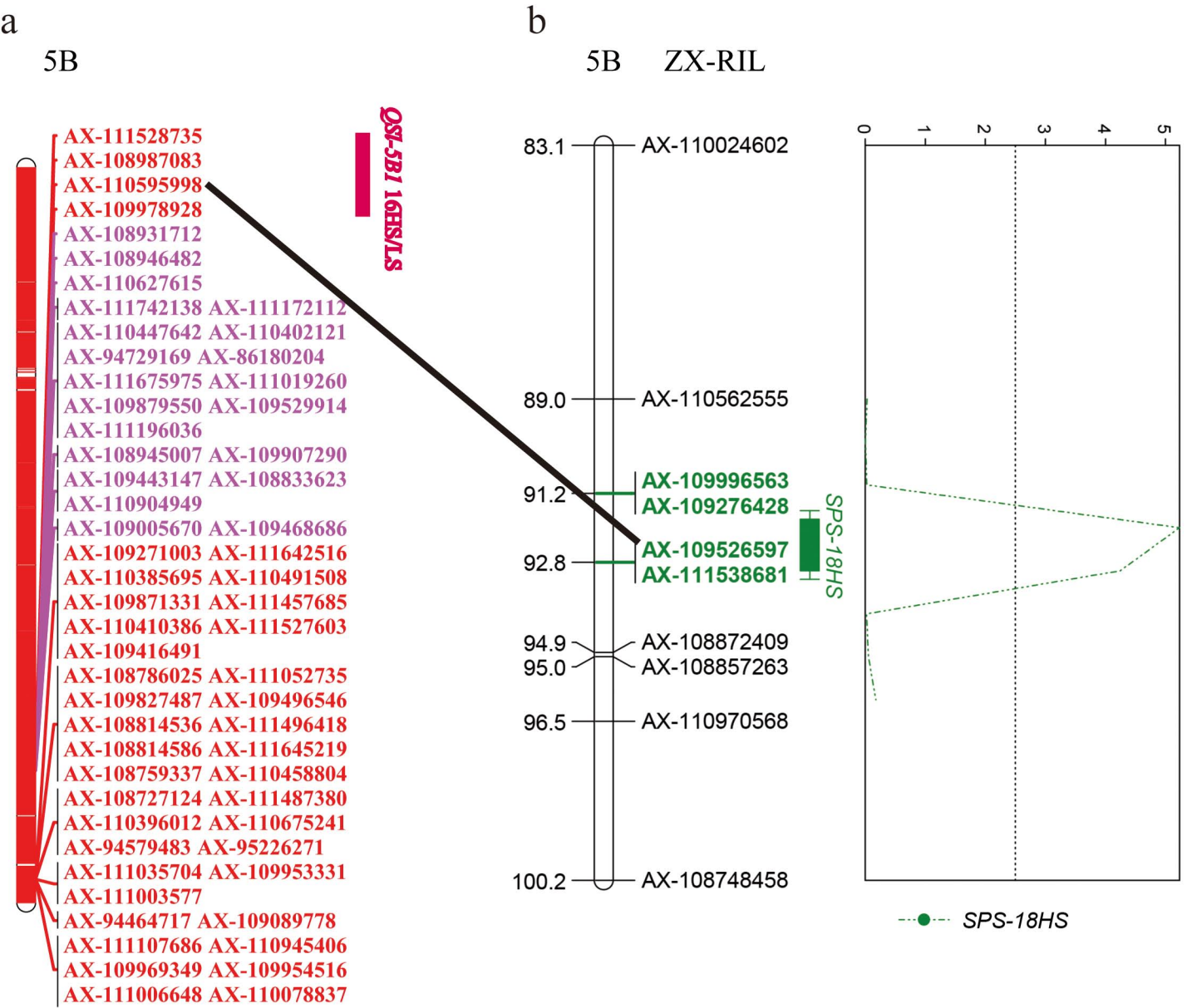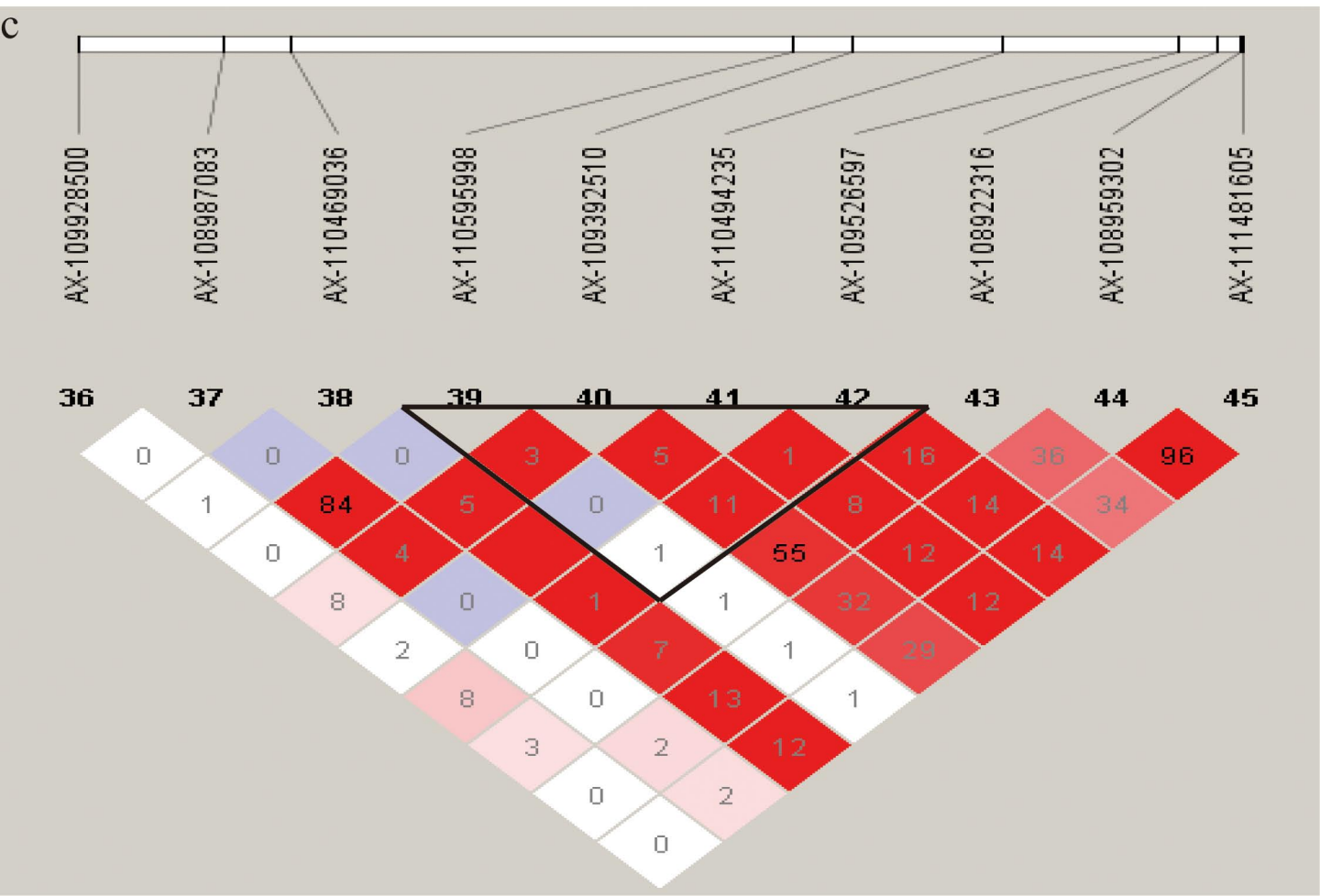

Supplement: Supplementary file 11 — Additional file 11: Figure S3. QTL position on chromosome 5B for spike length (SL) in the common wheat population and ZX-RIL population. (a) Chromosome location of Q-5B1 in the 191 common wheat accessions. (b) QTL position for SPS in RIL population of “Zhongmai 175×Xiaoyan 60”. (c) LD plot of the QSl-5B1 in the 191 common wheat accessions and the QTL for SPS in RIL population of “Zhongmai 175 × Xiaoyan 60”. [file 12870_2020_2799_MOESM11_ESM.pdf]

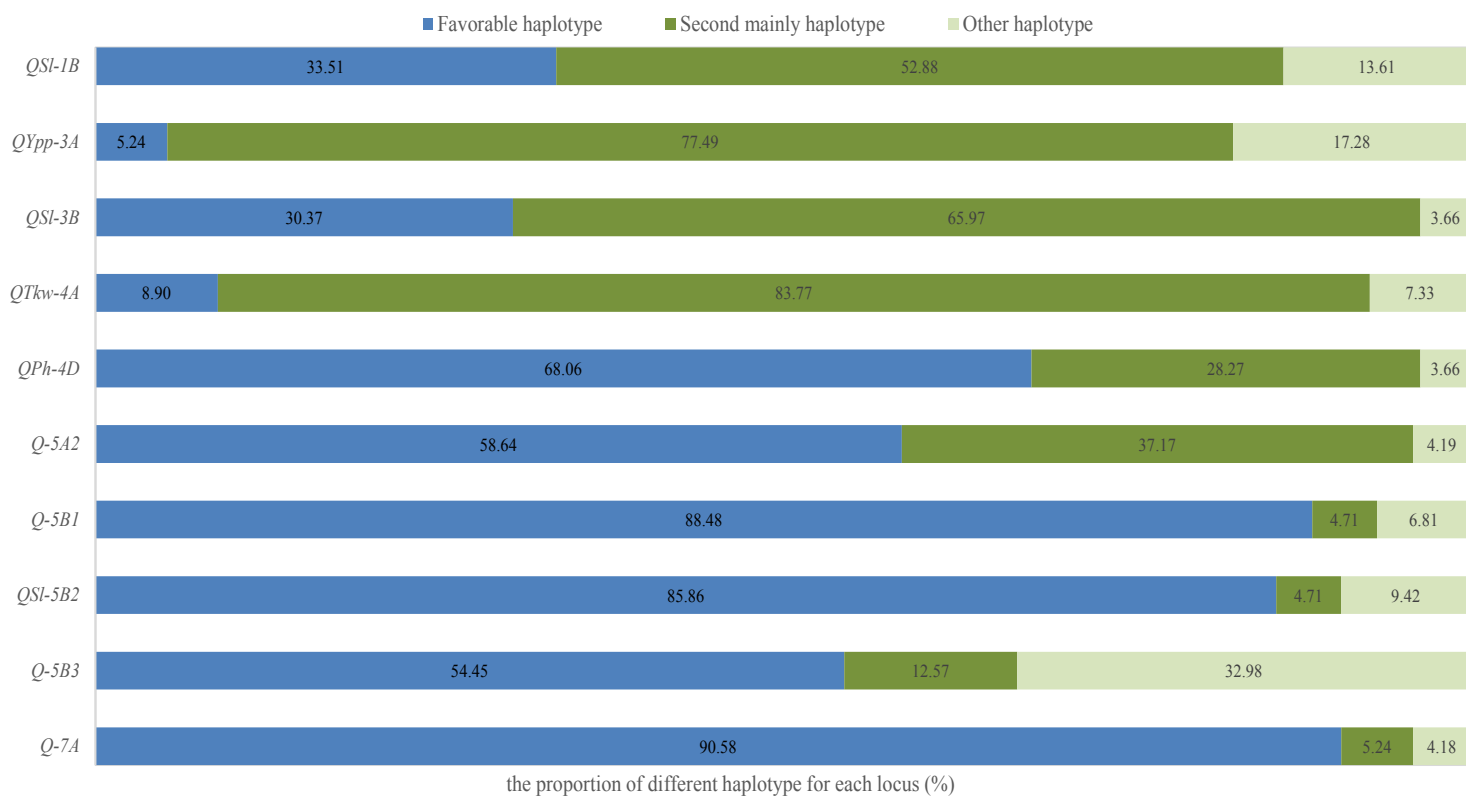

Supplement: Supplementary file 12 — Additional file 12: Figure S4. The proportion of different haplotypes for each locus. The percentage represented the proportion of haplotype in the 191 common wheat accessions. [file 12870_2020_2799_MOESM12_ESM.pdf]

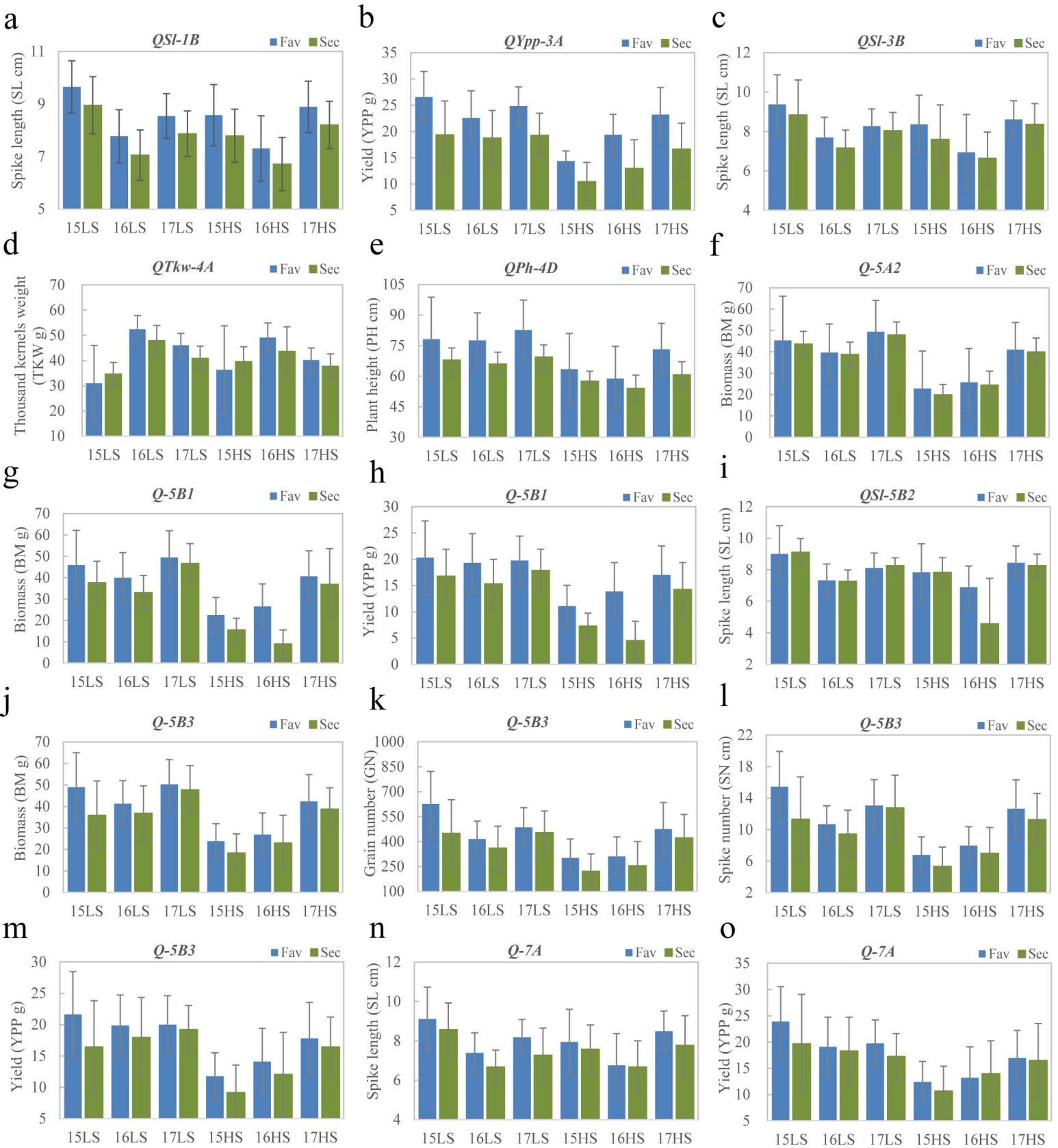

Supplement: Supplementary file 13 — Additional file 13: Figure S5. Haplotype analysis of the important loci. PH, plant height; SN, spike number; SL, spike length; GN, grain number; TKW, thousand kernels weight; YPP, yield per plot; BM, biological mass. LS, low salinity treatment; HS, high salinity treatment. [file 12870_2020_2799_MOESM13_ESM.pdf]

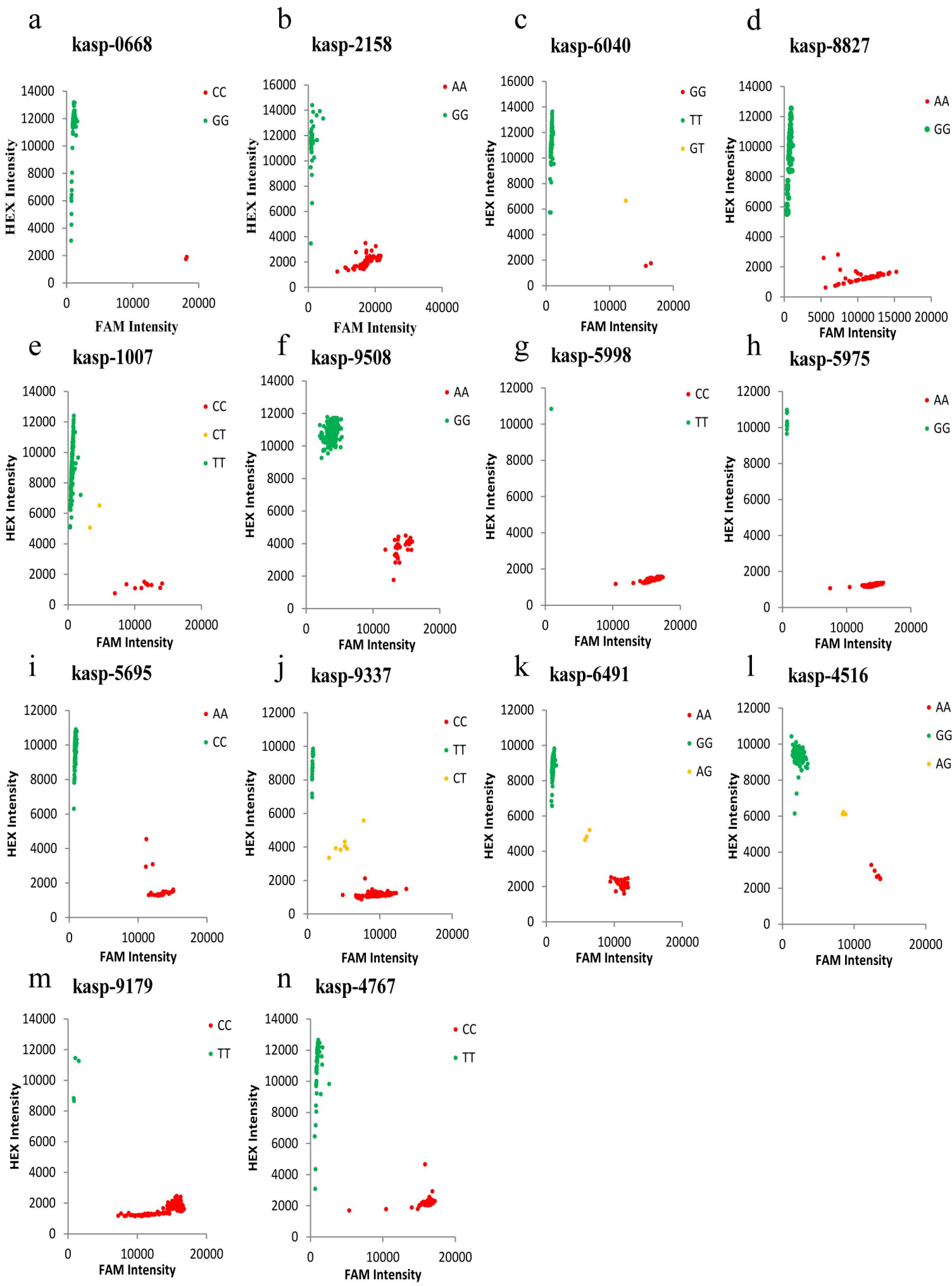

Supplement: Supplementary file 14 — Additional file 14: Figure S6. Scatter plots of the 14 KASP markers for nine key loci identified in the present study. Red dots indicate the accessions had the FAM-type allele; blue dots indicate the accessions had the HEX-type allele. [file 12870_2020_2799_MOESM14_ESM.pdf]
